# Tailoring CONSORT‐SPI to improve the reporting of smoking cessation intervention trials: An expert consensus study

**DOI:** 10.1111/add.16340

**Published:** 2023-09-18

**Authors:** Zoe Swithenbank, Alessio Bricca, Nicola Black, Jamie Hartmann Boyce, Marie Johnston, Neil Scott, Robert West, Ryan J. Courtney, Shaun Treweek, Susan Michie, Marijn de Bruin

**Affiliations:** ^1^ Public Health Institute Liverpool John Moores University Liverpool UK; ^2^ Research Unit for Musculoskeletal Function and Physiotherapy University of Southern Denmark Odense Denmark; ^3^ The Research Unit PROgrez Næstved‐Slagelse‐Ringsted Hospitals Slagelse Denmark; ^4^ National Drug and Alcohol Research Centre University of New South Wales Sydney Kensington Australia; ^5^ Nuffield Department of Primary Care Health Sciences University of Oxford Oxford UK; ^6^ Institute of Applied Health Sciences University of Aberdeen Aberdeen Scotland, UK; ^7^ Medical Statistics Team University of Aberdeen Aberdeen Scotland, UK; ^8^ Health Behaviour Research Centre, Department of Epidemiology and Public Health University College London London UK; ^9^ Health Services Research Unit University of Aberdeen Aberdeen Scotland UK; ^10^ Centre for Behaviour Change University College London London UK; ^11^ IQ Healthcare, Institute of Health Sciences Radboud University Medical Center Nijmegen the Netherlands

**Keywords:** expert consensus, interventions, RCTs, reporting, smoking cessation, trials

## Abstract

**Background and Aims:**

Inadequate reporting of smoking cessation intervention trials is common and leads to significant challenges for researchers. The aim of this study was to tailor CONSORT (Consolidated Standards of Reporting Trials)‐SPI (Social and Psychological Interventions) guidelines to improve reporting of trials of behavioural interventions to promote smoking cessation.

**Method:**

Informed by missing data from the IC‐SMOKE (Intervention and Comparison group support provided in SMOKing cEssation) systematic review project, this study used a multi‐stage Delphi process to examine which items could be added or modified to improve the reporting of smoking cessation trials. The first stage involved an on‐line survey of 17 international experts in smoking cessation and trial methodology voting on the importance of items for inclusion in the updated guidelines. This was followed by a face‐to‐face expert consensus meeting attended by 15 of these experts, where the final inclusion and exclusion of new items and modifications were agreed upon. A nine‐point Likert scale was used to establish consensus, with suggested modifications requiring agreement of 75% or more. Disagreements in the first stage were presented again at the second stage for discussion and a second round of voting. Only items which reached the threshold for agreement were included.

**Results:**

The experts agreed on the inclusion of 10 new items and the specification of 12 existing items. This included modifications that could apply to trials more widely (e.g. the rationale for the comparator), but also modifications that were very specific to smoking cessation trials (e.g. the reporting of smoking cessation outcomes).

**Conclusions:**

A Delphi study has developed a modified CONSORT‐SPI guideline (CONSORT‐SPI‐SMOKE) to improve the reporting of trials of behavioural interventions to promote smoking cessation.

## INTRODUCTION

Suboptimal trial reporting leads to a major loss of resources, hinders scientific progress and diminishes the quality of the evidence base to inform policy and practice [1, 2]. There is ample evidence of inadequate reporting of trials of behavioural interventions, including those evaluating smoking cessation interventions. For example, systematic reviews of behavioural intervention trials often fail to fully capture risk of bias features as well as the experimental and comparator interventions under study [3, 4, 5, 6]. For this reason, guidelines have been developed, such as the CONSORT statements (including an extension for social and psychological interventions: CONSORT‐SPI) [7] and the Template for Intervention Description and Replication (TIDieR [2]). Despite these two guidelines, under‐reporting remains common. For example, a recent study found no improvement in the reporting of smoking cessation interventions’ active ingredients over two decades, despite the ongoing call for improved reporting [8].

The IC‐Smoke project is a major methodological systematic review project of behavioural smoking cessation interventions (with and without pharmacological support) with detailed coding of methodological and intervention components, and author contact protocols when data are missing (https://osf.io/23hfv/). In the process of conducting this study, several important gaps and inconsistencies in the reporting of smoking cessation trials with regard to, for example, intervention content [8], the level of training of intervention providers, intervention implementation and the reporting of outcome data, were identified. Sometimes these items were required by CONSORT‐SPI, but CONSORT‐SPI did not specify the level of detail, e.g. ‘for each outcome, results for each group, and the estimated effect size and its precision (such as 95% confidence interval)’, leading to considerable variability in the level of detail provided by authors in trial manuscripts. Other items were not explicitly requested in CONSORT‐SPI but appeared to be important for understanding and synthesizing smoking cessation trials. Hence, while adherence to reporting guidelines is crucial and would have reduced missing information to some degree, it also appears the existing guidance is not always sufficiently detailed or comprehensive enough to include all the relevant information for behavioural smoking cessation interventions.

The aim of the current study was therefore to tailor CONSORT‐SPI to improve reporting of behavioural smoking cessation trials. Specifically, this study focused upon the two research questions related to improving the reporting of such trials: (1) which CONSORT‐SPI items can be further specified to improve the reporting of smoking cessation intervention trials and (2) which items should be added to CONSORT‐SPI to improve smoking cessation trials reporting?

## METHODS

### Design

A two‐stage modified Delphi process was adopted, which included first an on‐line survey and secondly a physical expert consensus meeting in Utrecht (Trimbos Institute, the Netherlands). In this consensus exercise we followed the Guidance for Developers of Health Research Reporting Guidelines [9] and the initial survey process was defined a priori although, as the initial phase was a feasibility assessment, the details for the in‐person meeting were developed in an iterative manner. As the primary research question and analysis plan were not pre‐registered, the results of this study should be considered exploratory.

### Procedure

The CONSORT‐SPI guidance was used as the backbone to this exercise. We examined whether an international group of smoking cessation experts judged it to be valuable to add or further specify items for the reporting of behavioural smoking cessation trials and, if so, what these modifications might be. The preparatory stage of this process was the IC‐SMOKE systematic review, the detailed methods and search strategy of which have been previously published [10, 11]. In this systematic review database, key areas of missing and inconsistent data were identified. Existing tools and guidance documents were also reviewed for the reporting of behavioural interventions for smoking cessation trials (including TIDieR [2], the Russell Standard [12] and *Addiction*’s Paper Authoring Tool [13]) to identify items and descriptions that could potentially complement CONSORT‐SPI. These items were presented to experts in an on‐line Delphi study (description below), during which experts could also propose additional items. The results from the on‐line Delphi were presented, discussed and voted on during the physical full‐day expert consensus meeting.

### Delphi survey

Seventeen experts from 10 organizations in seven countries were invited to take part in this study. The panel included 15 researchers from European countries and two from Australia. We also approached participants from the United States, Saudi Arabia and China. However (note that this was prior to ‘on‐line’ COVID‐times), they declined to take part given the travel distance. These included statisticians, trialists, experts in smoking cessation intervention research (trials, systematic reviews, smoking cessation policy), behaviour change experts and experts in smoking cessation. We made sure to include participants with practical experience of delivering smoking cessation. Some experts had also been involved in developing CONSORT and TiDieR statements (see Supporting information, Table S1). Experts were invited via e‐mail to take part in the Delphi survey and attend a face‐to‐face consensus meeting. Experts were selected upon recommendation from members of the project team and their extended networks.

Upon agreeing to participate, we asked experts to vote on‐line on the importance of further specifying or adding 11 items to CONSORT‐SPI, which we identified from the IC‐Smoke database and the literature review of existing tools and guidance documents for the reporting of behavioural interventions or smoking cessation trials. We followed the guidelines for voting laid out in the *COMET Handbook* [14]. Experts rated the items on a nine‐point Likert scale (1–3: not important; 4–6: important but not critical; 7–9: critical). Positive agreement was based on a score of 75% or more of the votes in the ‘critical’ category and fewer than 15% in the ‘not important’ category. Negative agreement was based on a score of 75% or more of the votes in the ‘not important’ category and fewer than 15% in the ‘critical’ category. Abstainers were counted as missing and are denoted in Table 1 below. All other scores were considered as disagreements and were discussed at the face‐to‐face consensus meeting. We also asked experts to add any additional items or specifications which they felt were important for the reporting of behavioural smoking cessation trials.

**TABLE 1:** Voting results: implementing the CONSORT‐SPI and developing smoking cessation‐specific items.

Label	Item description	When item was suggested	Voting (*n* voted critical for inclusion)	
	Additional item or specification for smoking cessation trials		Survey	Meeting
1a (1)	Specification: Include the design and primary research question or hypothesis	Suggested in original survey		0/15
1a (2)	Specification: In the case of a health economic evaluation, one may want to add ‘Health economic evaluation alongside a randomized trial’	Suggested during on‐line survey		0/15
1b	Specification: Include the setting of the trial	Suggested at face‐to‐face meeting		15/15
2a	Specification: Justification for the choice of intervention and comparator conditions	Suggested at face‐to‐face meeting		15/15
2b	Specification: Explanation for why the primary and secondary outcomes were chosen/explanation for any moderation or mediation analyses to be conducted	Suggested at face‐to‐face meeting		15/15
3a	Additional item: Justification and rationale for trial design, including timing of follow‐up measurements and the selection of the comparator	Suggested at face‐to‐face meeting		15/15
4a	Specification: Include whether participants were intending/not intending to quit smoking and distinction between use (one occasion/experimental /occasional/regular)	Suggested at face‐to‐face meeting		15/15
4b	Specification: Settings, locations and period when data were collected	Suggested during on‐line survey		0/15
4c	Additional item: How, where, when and by whom participants were recruited Specification of additional item: In particular, if motivation to quit was not an inclusion criterion, consideration of whether recruitment method would have selected people with higher levels motivation as they had to actively respond to trial recruitment messages (e.g. flyers, media advertisements) as opposed to being actively approached by a recruiter (e.g. opportunistic interventions at GP surgery)	Included in original survey	10/17	14/14
4d	Additional item: Recruitment cost	Suggested during on‐line survey		0/15
5	Amendment: The intervention for each group described in sufficient detail to allow replication Specification: Include training relevant training of those delivering intervention	Included in original survey	15/17	14/14
5b	Where all the intervention materials for each group (and any comparator, including usual care) and, in case of in‐person delivered interventions, training materials can be accessed Specification: Consider adding the checklist for reporting of comparator interventions in smoking cessation trials, when reporting on your control group(s) (see link OSF)	Included in original survey	10/17	14/14
5d	Additional item: What the rationale is behind selecting the comparator. In case of treatment‐as‐usual comparator, why these treatment‐as‐usual sites were recruited Specification: Evidence that ‘as usual’ treatment is indeed as usual Provide detailed definition about ‘treatment as usual’; i.e. potential intervention in the control group should be described with the same precision as in the intervention group	Included in original survey	12/17	15/15
6a	Specification: Consider the following criteria when reporting smoking cessation outcomes: • Description of what strategies or materials were used for outcome measurement • Type of biochemical verification (CO, cotinine, anabasine etc.), if any; • and justify if no biochemical assessment of smoking • Give definition of abstinence and cut‐point for verified abstinence • Report the starting point of timing of assessment (e.g. target quit date, onset of intervention) • Report outcomes at all time points assessed • Reduction in cigarettes per day as an outcome, if available and how this was measured	Included in original survey	15/17	15/15
7a	Specification: Ideally, provide several simple size estimation scenarios and justify the final choice	Suggested at face‐to‐face meeting		15/15
10	Specification: Include when participants were assigned to interventions	Suggested at face‐to‐face meeting		15/15
11a	Additional item: Specify for each outcome, whether and how outcome assessors were blinded to treatment assignment Specification: Note that in the cases of self‐reported smoking status, the participants themselves are the outcome assessors	Included in original survey	15/17^a^	14/15
12a	Specification: Specify analysis of primary outcome. Include how any absent biochemical data for those who self‐report continued smoking was handled	Suggested at face‐to‐face meeting		15/15
13b	Additional item: For each group, at least for the primary time‐point, specify non‐response, dropout and exclusions, together with reasons if possible (include sample descriptives for those excluded, declined participation, non‐responders, dropouts and those discontinuing the intervention)	Included in original survey	12/17	13/14
14b	Additional item: Why the trial ended, or recruitment was stopped before the pre‐specified sample size or follow‐up was achieved; or why trial recruitment was continued beyond the pre‐specified sample size or follow‐up duration.	Included in original survey	10/17	13/13
15	Additional item: Recommend minimum core data set with variables (e.g. nicotine dependence, motivation to quit, physical or mental illness, etc.) and measures Specification: Consider including: • Tobacco addiction (or dependence) using the Fagerstrom test (FTCD) or any other validated measure of tobacco addiction; • and/or number of cigarettes per day, time to first cigarette after awakening (more robust than total FTCD). • Any alternative nicotine delivery systems (ANDS) and other smoking cessation aids at baseline and follow‐up	Included in original survey	9/17^a^	13/14^a^
16b	Additional item: Report inappropriate administration of the intervention	Included in original survey	6/17	2/14
17a	Specification: Publish data set (or summary table) with: • Number of participants for primary outcomes including at each follow‐up • Number of participants who smoke, quit, non‐responders, drop out at each time‐point • Specify if outcome is: self‐report, objective or both	Suggested at face‐to‐face meeting		15/15
17c	Additional item: Availability and location of the statistical scripts for running the analysis over the outcome data set and of the statistical outputs. If these are not available, state why not	Included in original survey	7/17	14/15^a^
19	Additional item: Death and all other adverse outcomes should always be reported	Suggested during on‐line survey		0/15
20	Specification: Discuss outcomes of planned sensitivity analyses and how these attest or do not attest to the robustness of the findings	Suggested during online survey		13/15
25	Specification: Include any tobacco industry funding	Suggested at face‐to‐face meeting		14/14
26c	Specification: Include any gifts or other offerings, not just incentives. Include if medications or devices were given free of charge to participants, and if these were contingent on outcomes	Suggested at face‐to‐face meeting		14/14

*Note*: Items in green reached the accepted level of agreement for inclusion (75% or higher); items in red reached the accepted level of agreement for exclusion. Items in yellow did not reach consensus and were presented at the consensus meeting for further discussion.

Abbreviations: CO, carbon monoxide; FTCD, Fagerström Test for Cigarette Dependence; GP, general practitioner.

^a^ One voter abstained.

### Consensus meeting

Fifteen of the 17 experts attended the full‐day in‐person meeting (May 2019). The other two provided input by e‐mail. An experienced Chair who was not involved as an expert (Professor Diane Dixon, University of Aberdeen) led the meeting. The structure of the programme was to first present the results of the on‐line voting with high agreement (include and not include), to confirm that experts had no fundamental objections or additional arguments. Next, we focused upon items on which agreement had not been reached via the on‐line voting, including a summary of the on‐line comments in favour of or against including relevant items. This led to a discussion, after which the experts voted anonymously via an on‐line system [15] regarding whether items were either ‘critical’ or ‘not sufficiently important’ for inclusion in the new guidelines. On the day itself, experts proposed another 12 items to specify or add. These were also discussed and voted on.

### Follow‐up and completion of the guidelines

Following the meeting, the results and feedback were collated into the current manuscript(s), including a specified version of the CONSORT‐SPI (CONSORT‐SPI‐SMOKE). It was agreed that, rather than presenting our results as reporting requirements, these are additional recommended items and specifications that should help with the application of existing guidelines to behavioural smoking cessation trials. The final results consist of the CONSORT‐SPI checklist, with several additional items, changes of wording and the addition of specifications to promote the consistency of reporting of smoking cessation trials.

## RESULTS

**FIGURE 1:**
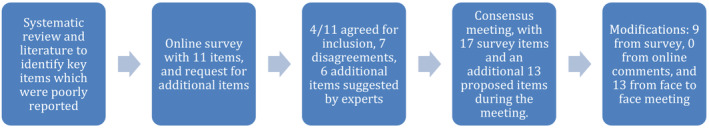
Overview of the steps in this study and the main results.

Figure 1 provides an overview of the results, which are discussed in more detail below. All proposed and discussed items are shown in Table 1, together with information on the stage at which they were included in the Delphi process and the results of each voting stage.

### Delphi survey

Of the 11 items identified in the literature review, there was agreement on four items (Table 1, numbers 5, 5d, 6a and 11a) and disagreement on seven items (Table 1, numbers 4c, 5b, 13b, 14b, 15, 16b, 17c). Experts proposed an additional six items via the survey. The authors were also asked to comment upon each of their choices. For example, for item 11a, the proposed addition was: specify for each outcome, whether and how outcome assessors were blinded to treatment assignment. Of the 17 voters, 15 voted that this item was critical (7–9 on the Likert scale), one voted important but not critical and one abstained. Comments to support the inclusion of this item were, for example: ‘this is really important and that all key risk of bias areas should be covered’ and ‘indeed, blinding should be conducted and reported well’. The only concerns were that this would be dependent upon the outcome measures used, as blinding would not be possible for self‐reported outcomes.

With regard to the seven disagreements, the comments were examined in detail and synthesized for the discussion at the expert meeting. An example is item 15, which requires a table showing pre‐specified baseline characteristics for participants per trial arm, such as socio‐economic variables. The proposed amendment was to include nicotine dependency and number of participants in each treatment arm that used e‐cigarettes and other smoking cessation aids (e.g. medication, nicotine patches) during follow‐up, where possible. Of the 17 voters, seven voted important but not critical, nine voted critical and one abstained. Key arguments in favour of this item included ‘important baseline variable to report’ and ‘abstinence measures should be defined a priori and should state if they only include combustible tobacco’. Of those who agreed that this was important but not critical, arguments primarily concerned standardized measures of nicotine dependence, with one expert suggesting: ‘I would prefer cigarettes per day over nicotine dependency’. Key arguments against the inclusion of this item were: ‘This is a catch 22. Where do you draw the line? Should we also be agreeing on other factors that moderate treatment effectiveness other than nicotine dependence? Therefore, should we be including a list of potential moderators that need to be reported in a baseline table?’

For an overview of the disagreements and the key arguments, please see the PowerPoint slides for the consensus meeting in the Supporting information.

### Consensus meeting results

For all four survey items with consensus (critical/not sufficiently relevant), experts agreed with that decision during the face‐to‐face consensus meeting. For the seven survey items with no consensus, the arguments in favour and against were presented and discussed. Finally, five of these were deemed critical by 75% or more and two not critical (< 75% critical). Next, the six additional items proposed by experts in the on‐line survey were discussed, but none of these were judged critical by 75% or more of the experts. This was in contrast with the 13 modifications suggested by experts on the day itself, all of which were voted as critical by 75% or more of the experts. In total, there was agreement on eight new items and 14 specifications to existing items. The final version of the CONSORT‐SPI‐SMOKE is shown below in Table 2.

**TABLE 2:** Modified CONSORT‐SPI with recommendations for reporting smoking cessation trials: CONSORT‐SPI‐SMOKE.

Section	Item	CONSORT‐SPI 2018 + modifications (*in italics*)	For smoking cessation intervention trials, it is recommended to
Title and abstract see also extension for economic evaluations of health interventions (if relevant)			
	1b	Structured summary of trial design, methods, results and conclusions (for specific guidance see CONSORT for Abstracts)^§^ *Refer to CONSORT extension for social and psychological intervention trial abstracts (see below)*	Include the setting of the trial
Introduction			
Background and objectives	2a	Scientific background and explanation of rationale^§^	Justification for the choice of intervention and comparator conditions
	2b	Specific objectives or hypotheses^§^ If pre‐specified, how the intervention was hypothesized to work	Include: Explanation for why the primary and secondary outcomes were chosen Explanation for any moderation or mediation analyses to be conducted
Methods			
Trial design	3a	Description of trial design (such as parallel, factorial), including allocation ratio^§^ *Justification and rationale for trial design, including timing of follow‐up measurements and the selection of the comparator* If the unit of random assignment is not the individual, please refer to CONSORT for cluster randomized trials [33]	
Participants	4a	Eligibility criteria for participants^§^ When applicable, eligibility criteria for settings and those delivering the interventions	Include whether participants were intending/not intending to quit smoking
	4c	*How, where, when and by whom participants were recruited*	In particular, if motivation to quit was not an inclusion criterion, consideration of whether recruitment method would have selected people with higher levels motivation as they had to actively respond to trial recruitment messages (e.g. flyers, media advertisements) as opposed to being actively approached by a recruiter (e.g. opportunistic interventions at GP surgery)
Interventions	5	The interventions for each group with sufficient details to allow replication, including how and when they were actually administered^§^ *The interventions for each group (including any comparator, including usual care) are described in sufficient detail to allow replication, including what was provided, why, how, by whom, when and how much, and where* *A template for reporting intervention description and replication exists for this CONSORT 2010 item (TIDieR)* [2]	Include training relevant training of those delivering intervention. Some pragmatic trials will test against usual care and may not be able to describe usual care very well. In some contexts, this is acceptable, but an explanation should be provided
	5b	Where other informational materials about delivering the intervention can be accessed, such as intervention protocols, training manuals or other materials (e.g. worksheets and websites) *Where all the intervention materials for each group (and any comparator, including usual care) and, in case of in‐person delivered interventions, training materials can be accessed*	Consider adding the checklist for reporting of comparator interventions in smoking cessation trials, when reporting on your control group(s) (see link OSF)
	5d	*What the rationale is behind selecting the comparator. In case of treatment‐as‐usual comparator, why these treatment‐as‐usual sites were recruited*	Evidence that ‘as usual’ treatment is indeed as usual. Provide detailed definition about ‘treatment as usual’; i.e. potential intervention in the control group should be described with the same precision as in the intervention group
Outcomes	6a	Completely defined pre‐specified outcomes, including how and when they were assessed^§^	Consider the following criteria when reporting smoking cessation outcomes: Description of what strategies or materials were used for outcome measurement Type of biochemical verification (CO, cotinine, anabasine etc.), if any and justify if no biochemical assessment of smoking Give definition of abstinence and cut‐point for verified abstinence Report the starting‐point of timing of assessment (e.g. target quit date, onset of intervention) Report outcomes at all time‐points assessed Reduction in cigarettes per day as an outcome, if available and how this was measured
Sample size	7a	How sample size was determined^§^	Ideally, provide several simple size estimation scenarios and justify the final choice.
Implementation	10	Who generated the random allocation sequence, who enrolled participants and who assigned participants to interventions^§^	Include when participants were assigned to interventions
Awareness of assignment	11a	Who was aware of intervention assignment after allocation (for example, participants, providers, those assessing outcomes) and how any masking was conducted *Specify for each outcome, whether and how outcome assessors were blinded to treatment assignment*	Note that in the cases of self‐reported smoking status, the participants themselves are the outcome assessors
Analytical methods	12a	Statistical methods used to compare group outcomes^§^ How missing data were handled, with details of any imputation method	Specify analysis of primary outcome Include how any absent biochemical data for those who self‐report continued smoking were handled
Results			
	13b	For each group, losses and exclusions after randomization, together with reasons *For each group, at least for the primary time‐point, specify non‐response, dropout and exclusions, together with reasons if possible (include sample descriptives of those excluded, declined participation, non‐responders, dropouts and those discontinuing the intervention)*	
	14b	Why the trial ended or was stopped *Why the trial ended or recruitment was stopped before the pre‐specified sample size or follow‐up was achieved; or why trial recruitment was continued beyond the pre‐specified sample size or follow‐up duration*	
Baseline data	15	A table showing baseline characteristics for each group^§^ Include socio‐economic variables where applicable *Recommend minimum core data set with variables (e.g. nicotine dependence, motivation to quit, physical or mental illness, etc.) and measures*	Consider including: Tobacco addiction (or dependence) using the Fagerstrom test (FTCD) or any other validated measure of tobacco addiction and/or number of cigarettes per day, time to first cigarette after awakening (more robust than total FTCD) Any alternative nicotine delivery systems (ANDS) and other smoking cessation aids at baseline and follow‐up
Outcomes and estimation	17a	For each outcome, results for each group, and the estimated effect size and its precision (such as 95% confidence interval)^§^ Indicate availability of trial data	Publish data set (or summary table) with: Number of participants for primary outcomes including at each follow‐up Number of participants who smoke, quit, non‐responders, dropout at each time‐point Specify if outcome is: self‐report, objective or both, including whether the objective assessment was conditional on the self‐reported In a format that allows for analyzing individual patient trajectories across multiple measurements, if applicable Include algorithm for deriving outcomes when time‐points are missing, for example: ‘If a participant did not attend all follow‐up appointments, we used data from the closest available timepoint to 6 months as the primary outcome’ If it is not possible to include all information within the paper, explain where this information can be accessed
	17c	*Availability and location of the statistical scripts for running the analysis over the outcome data set and of the statistical outputs. If these are not available, state why not*	Where possible. While this is good practice, it is not expected that data would be cleaned and presented in a user‐friendly format if this would be burdensome
Discussion			
Limitations	20	Trial limitations, addressing sources of potential bias, imprecision and, if relevant, multiplicity of analyses *Discuss outcomes of planned sensitivity analyses and how these attest (or do not attest) to the robustness of the findings*	
Important information			
Declaration of interests	25	Sources of funding and other support; role of funders Declaration of any other potential interests	Include any tobacco industry funding
Stakeholder involvement	26c	Incentives offered as part of the trial	Include any gifts or other offerings, not just incentives. Include if medications or devices were given free of charge to participants, and if these were contingent on outcomes

*Note*: § an extension for this item is available.

Abbreviations: CO, carbon monoxide; CONSORT, Consolidated Standards of Reporting Trials; FTCD, Fagerström Test for Cigarette Dependence; GP, general practitioner; IC‐SMOKE, Intervention and Comparison group support provided in SMOKing cEssation); SPI, Social and Psychological Interventions; TIDieR, Template for Intervention Description and Replication.

A key theme in the discussions at the face‐to‐face meeting included the need to balance the desire to collect comprehensive data with the burden this could place on trialists, in terms of both data collection and reporting of, for example, statistical scripts and data sets (which involves cleaning and presenting the data in a format usable by others). As one expert explained: ‘if you ask for too much, you won’t get the essentials’. This was balanced against the importance of transparency in trial reporting for all study groups, and the importance of reproducing and additional analyses as part of a cumulative science.

Another theme was emphasizing the importance of explanation and justification of trial design choices (their rationale), which should provide a clearer understanding of the way in which trials are designed and implemented. An example is the selection of the comparator in the trial, which should be based on a clear rationale [16]. The rationale for certain key design choices was judged to be generally poorly reported, despite being an important aspect of trial design, while inclusion of this in reporting recommendation is not adding an additional data collection or reporting burden on trialists.

A third theme in the discussion was the availability of all intervention materials (both for the experimental and comparator arms). Despite CONSORT‐SPI calling for all intervention materials to be made available, experts felt that this was often not done: ‘I think it’s scientifically invalid and unethical research to publish about an intervention that can’t be replicated. Supposing it’s something that’s damaging, and you don’t know what it was… that seems seriously unethical’. As one expert commented, this applies to all treatment arms—not just the experimental intervention: ‘to add ‘and any comparator’ (as described in CONSORT‐SPI) is not a novel thing, it’s an explicit reminder that it isn’t just the experimental intervention that this applies to’. Another part of this discussion focused upon whether this would be possible without sharing copyrighted or otherwise sensitive information. Again, a pragmatic approach was agreed upon, with links or appendices being used rather than publishing all intervention materials alongside a publication, using the validated comparator intervention checklist for all trial arms [17] and otherwise include an explanation as to why these materials or scripts would not be made available.

It was also discussed that wherever relevant, other existing tools should be utilized. For example, instead of developing new items, experts proposed adding several references to other CONSORT extensions and the TIDieR checklist. Another discussion point was flexibility, as not every item will be relevant for or appropriate for every trial: ‘some items are essential for every trial, and some things are for some’. The tailored version therefore distinguishes between the existing CONSORT‐SPI items, and the modifications for smoking cessation proposed in this study that are described as ‘recommended’ and some as ‘if applicable’.

## DISCUSSION

A multi‐stage Delphi study including international experts on smoking cessation, trial design and methodology resulted in 22 recommendations for additions or amendments to the CONSORT‐SPI checklist, in order to address key issues in the reporting of smoking cessation trials identified in a systematic review and expert consultation. A tailored version of the CONSORT‐SPI is proposed for this purpose: the CONSORT‐SPI‐SMOKE.

Although CONSORT‐SPI offers a strong foundation for trial reporting, there are several aspects which appear to be covered in insufficient detail for the reporting of smoking cessation trials. For example, for outcome reporting the CONSORT‐SPI guidance is too generic, so that even when authors follow CONSORT‐SPI there may be substantial variability between trials in the type of information reported—information that is important for comparing and synthesizing smoking cessation trials. The use of different abstinence measures (point‐prevalence or continuous), type of objective assessment (e.g. cotinine, anabasine), whether these were assessed among all participants or only among people who self‐reported to have quit smoking, across all time‐points needs to be reported. A more generic reporting tool such as CONSOR‐SPI cannot capture all such intricacies for each SPI domain.

The study also identified several recommended amendments for SPI interventions more generally. For example, it was agreed that authors should include a justification for the choice of intervention and comparator conditions in the trial report. The latter, in particular, is often not done in trial reports, despite the choice of comparator being directly relevant to the research question. Poor reporting of comparators may obscure results from meta‐analyses of SPI trials [8, 18]. Another example of an item that is relevant to SPI trials more generally is to provide data on how, where, when and by whom participants were recruited to the trial, as this is potentially relevant to trial replication and intervention implementation, but also allows for conducting meta‐analyses on factors that enhance or hinder recruitment to trials [11]. These changes could be considered for inclusion in a next iteration of the CONSORT‐SPI guidance.

### Strengths and limitations

A strength of the current study is that it builds upon the work that has already been conducted on formulating guidelines such as CONSORT‐SPI, and that renowned experts, with different backgrounds from across the world, were included to agree on what amendments would make this guidance even more useful for smoking cessation trials. Additionally, experts had deliberately presented the amendment as suggested additions rather than requirements, as the burden on trialists and study participants is already high: the amendments presented here aim to make trial reporting easier without having to collect much more additional data. There are some limitations to the study; while there are additional items that could have been included to ensure that the guidelines were as relevant and useful as possible to as many trialists as possible, the experts prioritized items accordingly. An important limitation is the geographical reach of the expert panel (mainly Europe). Although attempts were made to increase the diversity of the panel, we acknowledge that the expert panel was lacking in ethnic and economic diversity, and as such the findings may have limited applicability beyond European countries. Participants were invited from Asia and North America, although many declined due to travel distance. The panel was also lacking in practical and lived experience perspectives, which may have increased the real‐world applicability of these recommendations. The a priori development of the Delphi process can be perceived as both a strength and a limitation of the study. The initial process was defined prior to the beginning of the study, but the following process was developed iteratively after feedback and consensus from the expert panel that the proposed modification to the CONSORT‐SPI was both feasible and necessary.

## CONCLUSION AND RECOMMENDATIONS

In conclusion, via expert consensus exercise, an extension of the CONSORT‐SPI for reporting smoking cessation trials was created (CONSORT‐SPI‐SMOKE). Adherence to these recommendations can help address key gaps in the reporting of smoking cessation trials, identified via systematic reviews and expert consensus.

## AUTHOR CONTRIBUTIONS

**Zoe Swithenbank:** Conceptualization (supporting); methodology (supporting); writing—original draft (lead); writing—review and editing (lead). **Alessio Bricca:** Conceptualization (supporting); methodology (supporting); writing—review and editing (equal). **Nicola Black:** Conceptualization (supporting); writing—review and editing (supporting). **Jamie Hartmann Boyce:** Conceptualization (supporting); writing—review and editing (supporting). **Marie Johnston:** Conceptualization (supporting); writing—review and editing (supporting). **Neil Scott:** Conceptualization (supporting); writing—review and editing (supporting). **Robert West:** Conceptualization (supporting); writing—review and editing (supporting). **Ryan J. Courtney:** Conceptualization (supporting); writing—review and editing (supporting). **Shaun Treweek:** Conceptualization (supporting); writing—review and editing (supporting). **Susan Michie:** Conceptualization (supporting); writing—review and editing (supporting). **Marijn de Bruin:** Conceptualization (lead); funding acquisition (lead); methodology (lead); writing—original draft (supporting); writing—review and editing (supporting).

## DECLARATION OF INTERESTS

The authors have no competing interests to declare.

## Supporting information


**Appendix S1.** Expert details.

**Data S1.** Supporting Information.

## Data Availability

The data that support the findings of this study are available on request from the corresponding author. The data are not publicly available due to privacy or ethical restrictions.
