# Supplementary material for: Tailoring CONSORT‐SPI to improve the reporting of smoking cessation intervention trials: An expert consensus study
Source: Addiction. 2023 Sep 18;119(2):225–35. doi: 10.1111/add.16340 (PMC10952324; doi:10.1111/add.16340)
Supplement: Supplementary file 1 — Appendix S1. Expert details. [file ADD-119-225-s002.docx]

**Appendix 1: Expert details**

| **Name** | **Male/female** | **Country** | **Expertise** | **Academic / other** | **Affiliations at time of involvement** |
| --- | --- | --- | --- | --- | --- |
| Prof. Paul Aveyard | Male | UK | Behavioural medicine | Academic | *Professor of Behavioural Medicine, University of Oxford*  *Coordinating editor Cochrane Tobacco Cessation* |
| Ass. Prof. Ivan Berlin | Male | Switzerland | Pharmacology | Academic / clinical | *Associate Professor in Clinical Pharmacology, at the Pitié-Salpêtrière Hospital, Paris and Policlinique Médical Universitaire, Lausanne, Switzerland* |
| Dr Nicola Black | Female | Australia | Health psychology | Academic | *Research Fellow, National Drugs and Alcohol Research Centre, Sydney* |
| Dr Alessio Bricca | Male | Italy | Health sciences | Academic | *Research Fellow, University of Aberdeen* |
| Dr Ryan Courtney | Male | Australia | Health behaviour science | Academic | *Research Fellow in health behavior science at University of New South Wales, National Drug and Alcohol Research Centre, Sydney.* |
| Prof. Marijn de Bruin | Male | Netherlands | Health psychology | Academic | *Professor of Health Psychology, University of Aberdeen; Hypatia Fellow, Radboud University Medical Centre at IQ Healthcare, Netherlands* |
| Dr Jamie Hartmann-Boyce | Female | UK | Health behaviours | Academic | *Senior Researcher in Health Behaviours, University of Oxford* |
| Prof. Marie Johnston | Female | UK | Health psychology | Academic | *Professor Emeritus of Health Psychology at the University of Aberdeen* |
| Prof. Eva Kralikova | Female | Czech Republic | Tobacco dependence | Academic | *Professor, Centre for Tobacco-Dependent of the 3^rd^ Medical Department, and Institute of Hygiene and Epidemiology, First Faculty of Medicine, Charles University, Czech Republic, and the General University Hospital in Prague* |
| Dr Andy McEwen | Male | UK | Practical smoking cessation | Treatment | *Director of the National Centre for Smoking Cessation and Training* |
| Prof. Susan Michie* | Female | UK | Health psychology | Academic | *Professor of Health Psychology and Director of the Centre for Behaviour Change at University College London* |
| Dr Neil Scott | Male | UK | Medical statistics | Academic | *Research Fellow, Medical Statistics, University of Aberdeen* |
| Prof. Filip Smit | Male | Netherlands | Evidence based public health | Academic | *Professor of evidence-based public mental health, Department of Epidemiology and Biostatistics and the Department of Clinical, Neuro and Developmental Psychology at Amsterdam Public Health Research Institute, University Medical Centers Amsterdam, location VUmc. Chief Scientist at the Trimbos Institute in Utrecht.* |
| Ms Zoe Swithenbank | Female | UK | Public Health | Academic | *Research Assistant, University of Aberdeen* |
| Dr Serena Tonstad* | Female | Norway | Public health / preventative cardiology | Academic / clinician | *Head physician at the Preventive Cardiology section, Department of Endocrinology, Obesity and Preventive Medicine at Oslo University Hospital, Oslo, Norway* |
| Prof. Shaun Treweek | Male | UK | Trial design and health services research | Academic | *Chair in Health Services Research, University of Aberdeen* |
| Prof. Robert West | Male | UK | Health psychology | Academic | *Professor of Health Psychology, University College London* |

*Completed the online survey but were not able to attend the face-to-face meeting.
