# Supplementary material for: Tailoring CONSORT‐SPI to improve the reporting of smoking cessation intervention trials: An expert consensus study
Source: Addiction. 2023 Sep 18;119(2):225–35. doi: 10.1111/add.16340 (PMC10952324; doi:10.1111/add.16340)
Supplement: Supplementary file 2 — Data S1. Supporting Information. [file ADD-119-225-s001.pptx]

## Slide 1
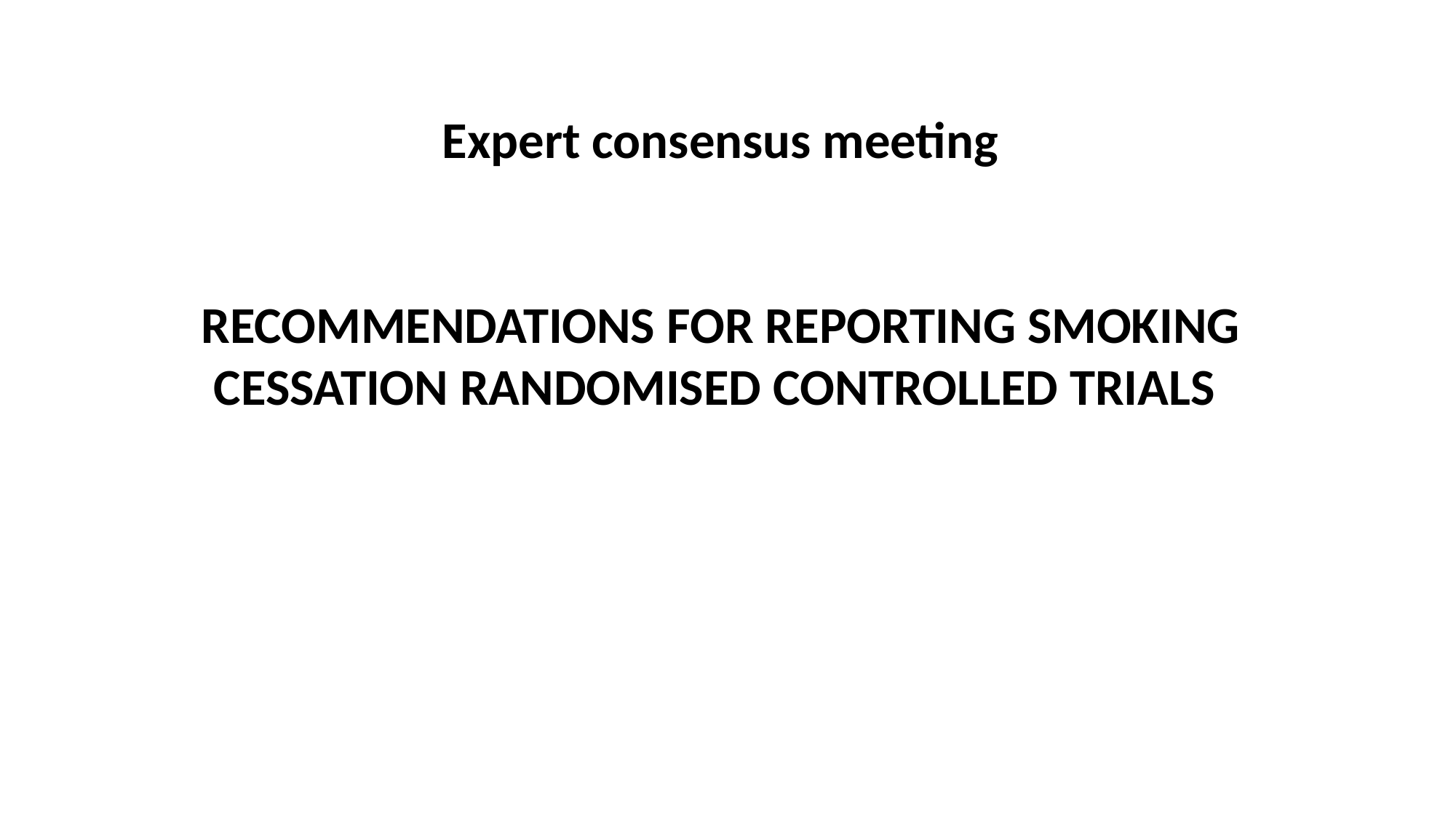

Expert consensus meeting
RECOMMENDATIONS FOR REPORTING SMOKING CESSATION RANDOMISED CONTROLLED TRIALS
1

## Slide 2
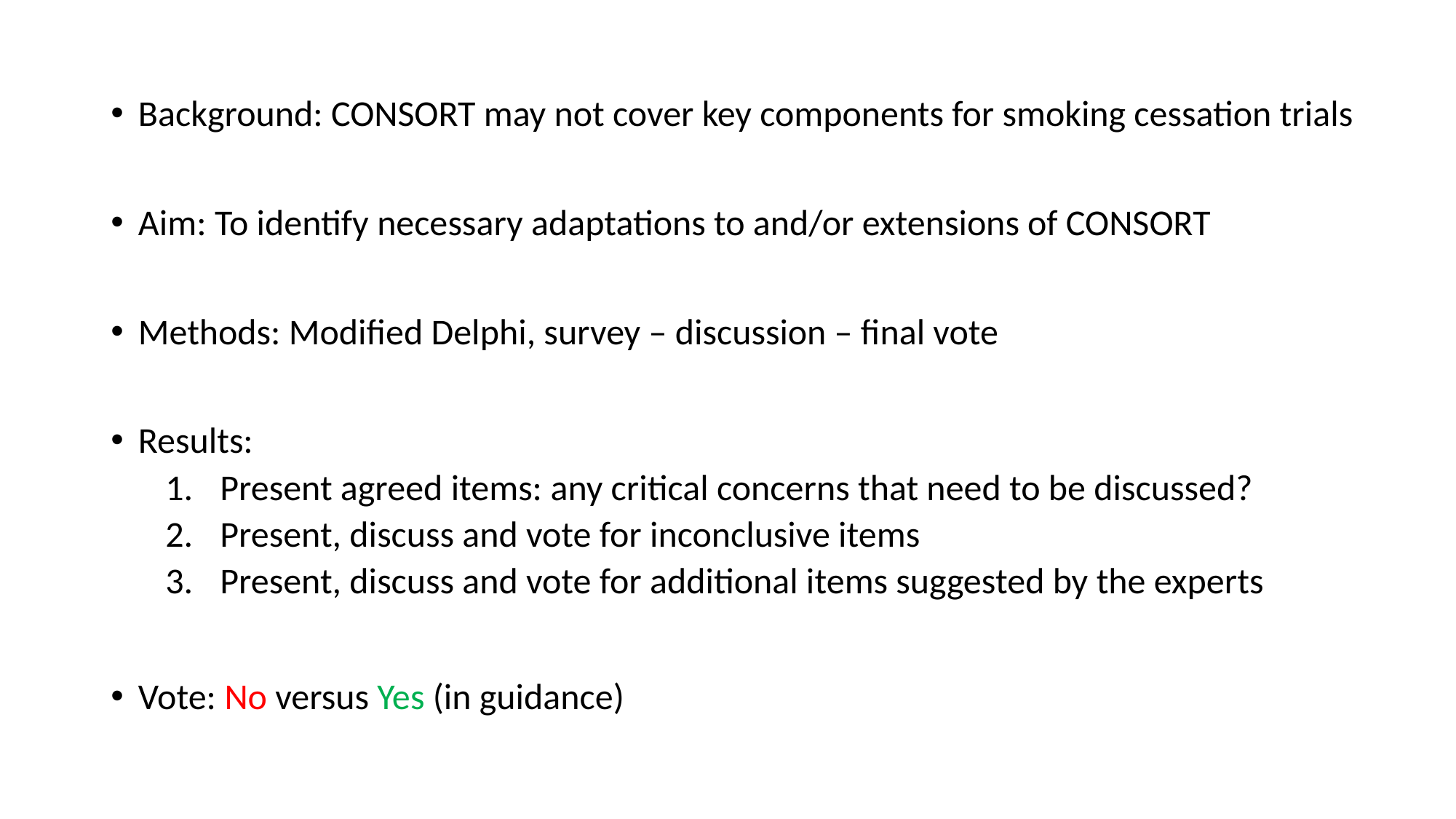

Background: CONSORT may not cover key components for smoking cessation trials
Aim: To identify necessary adaptations to and/or extensions of CONSORT
Methods: Modified Delphi, survey – discussion – final vote
Results:
Present agreed items: any critical concerns that need to be discussed?
Present, discuss and vote for inconclusive items
Present, discuss and vote for additional items suggested by the experts
Vote: No versus Yes (in guidance)

## Slide 3
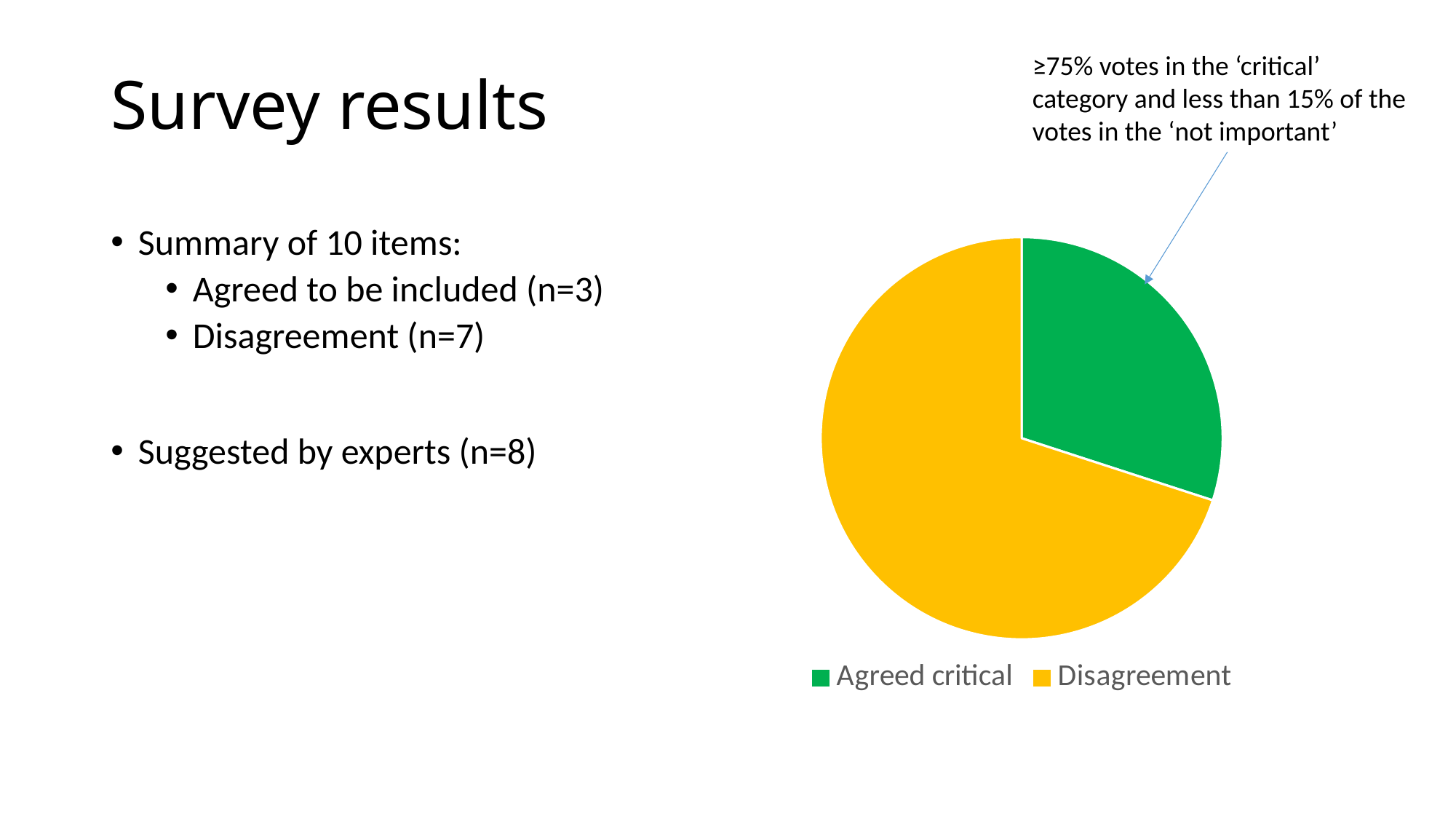

≥75% votes in the ‘critical’ category and less than 15% of the votes in the ‘not important’
# Survey results
Summary of 10 items:
Agreed to be included (n=3)
Disagreement (n=7)
Suggested by experts (n=8)
### Chart
| Category | Sales |
|---|---|
| Agreed critical | 3.0 |
| Disagreement | 7.0 |

## Slide 4
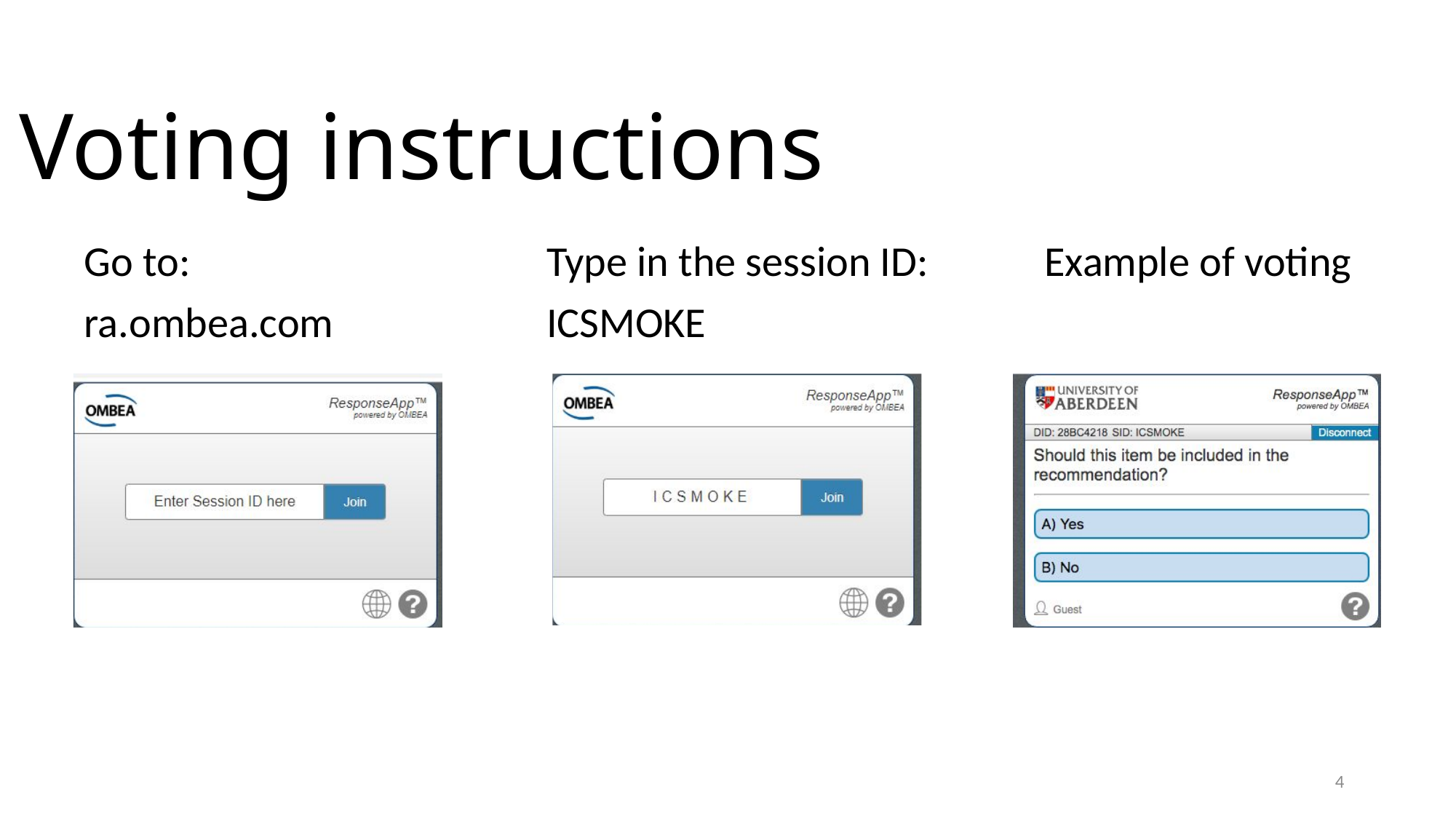

Voting instructions
Example of voting
Go to:
ra.ombea.com
Type in the session ID:
ICSMOKE
4

## Slide 5
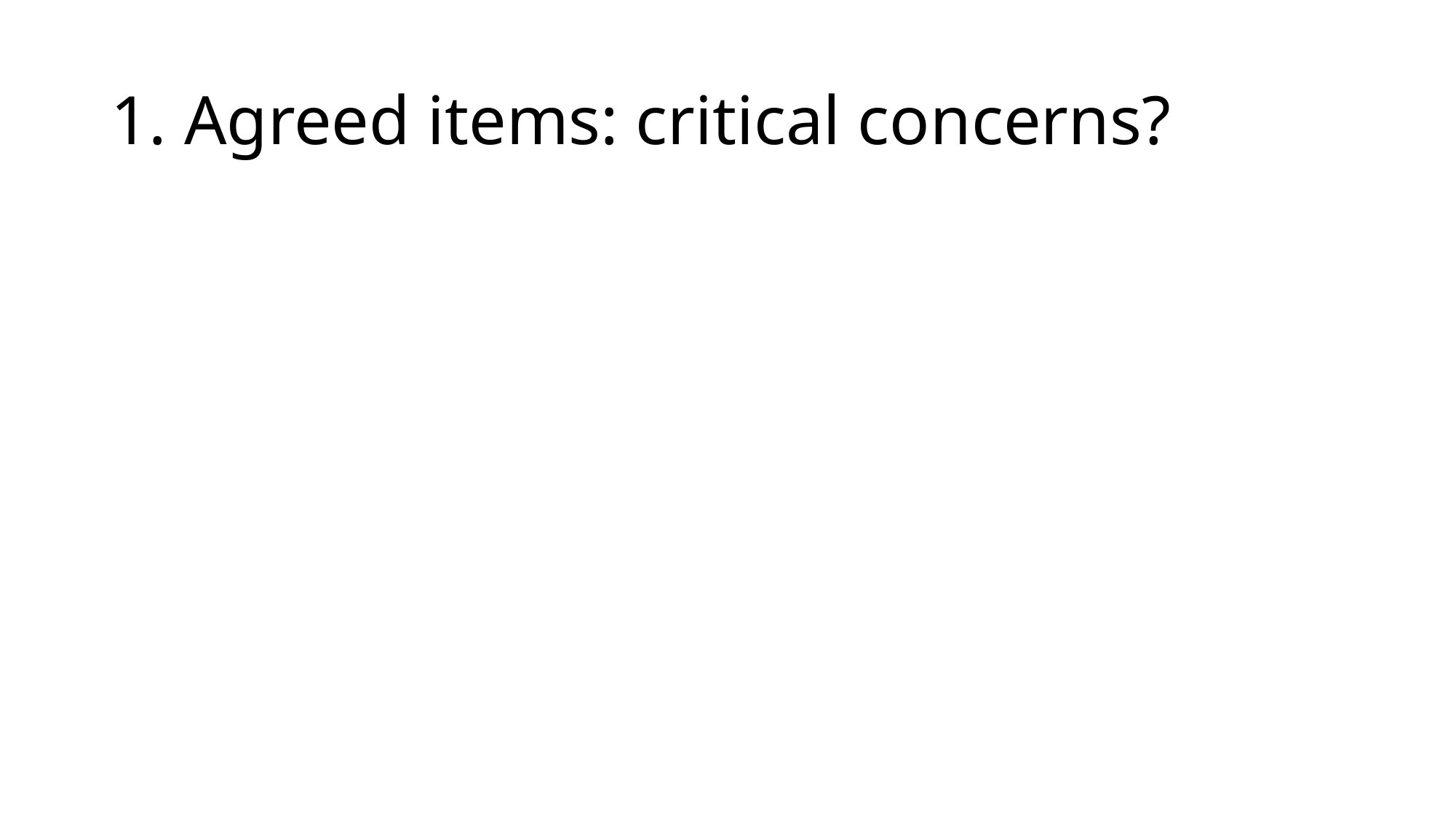

# 1. Agreed items: critical concerns?

## Slide 6
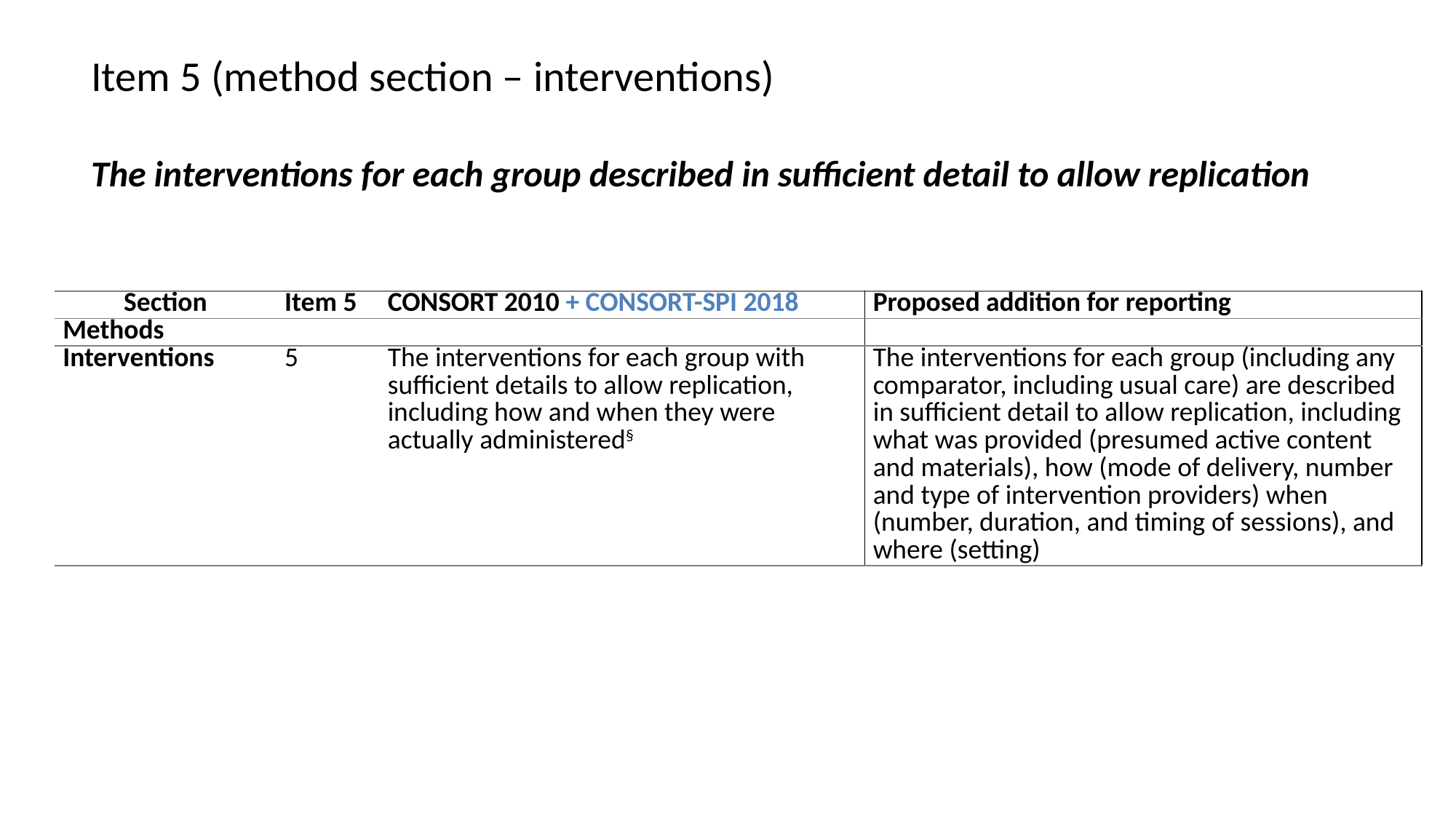

Item 5 (method section – interventions)
The interventions for each group described in sufficient detail to allow replication
| Section | Item 5 | CONSORT 2010 + CONSORT-SPI 2018 | Proposed addition for reporting |
| --- | --- | --- | --- |
| Methods | | | |
| Interventions | 5 | The interventions for each group with sufficient details to allow replication, including how and when they were actually administered§ | The interventions for each group (including any comparator, including usual care) are described in sufficient detail to allow replication, including what was provided (presumed active content and materials), how (mode of delivery, number and type of intervention providers) when (number, duration, and timing of sessions), and where (setting) |

## Slide 7
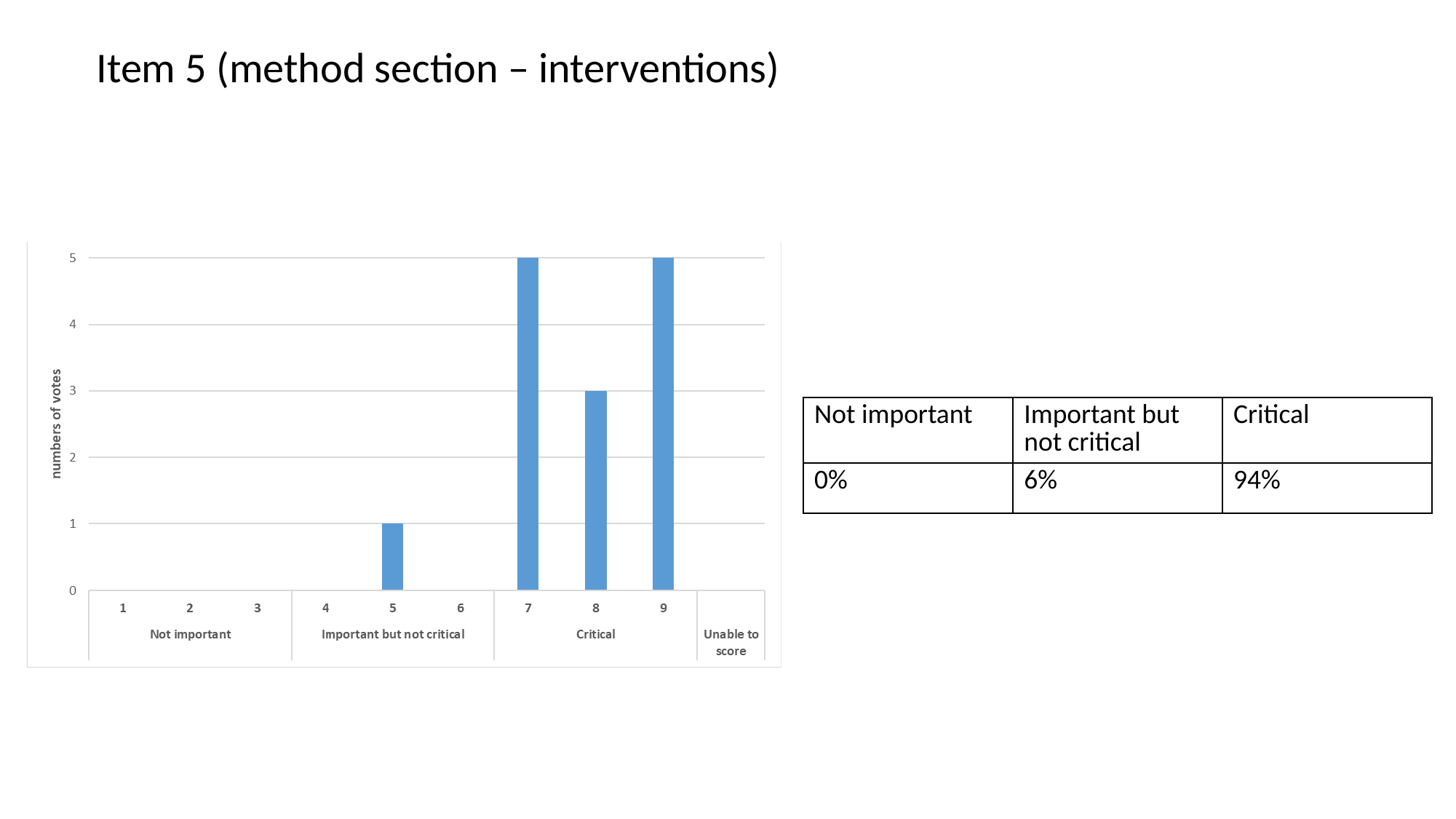

Item 5 (method section – interventions)
| Not important | Important but not critical | Critical |
| --- | --- | --- |
| 0% | 6% | 94% |

## Slide 8
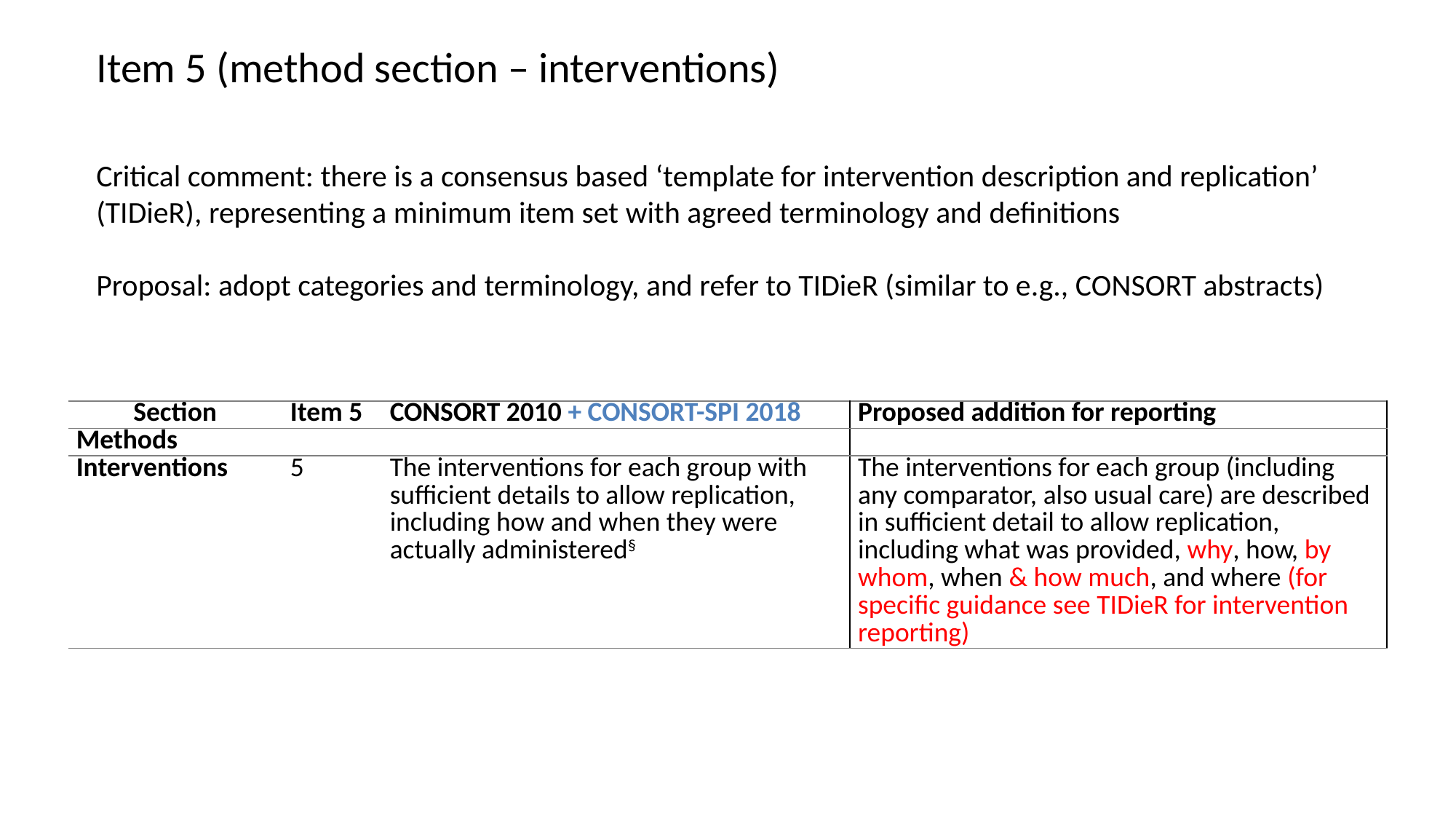

Item 5 (method section – interventions)
Critical comment: there is a consensus based ‘template for intervention description and replication’ (TIDieR), representing a minimum item set with agreed terminology and definitions
Proposal: adopt categories and terminology, and refer to TIDieR (similar to e.g., CONSORT abstracts)
| Section | Item 5 | CONSORT 2010 + CONSORT-SPI 2018 | Proposed addition for reporting |
| --- | --- | --- | --- |
| Methods | | | |
| Interventions | 5 | The interventions for each group with sufficient details to allow replication, including how and when they were actually administered§ | The interventions for each group (including any comparator, also usual care) are described in sufficient detail to allow replication, including what was provided, why, how, by whom, when & how much, and where (for specific guidance see TIDieR for intervention reporting) |

## Slide 9
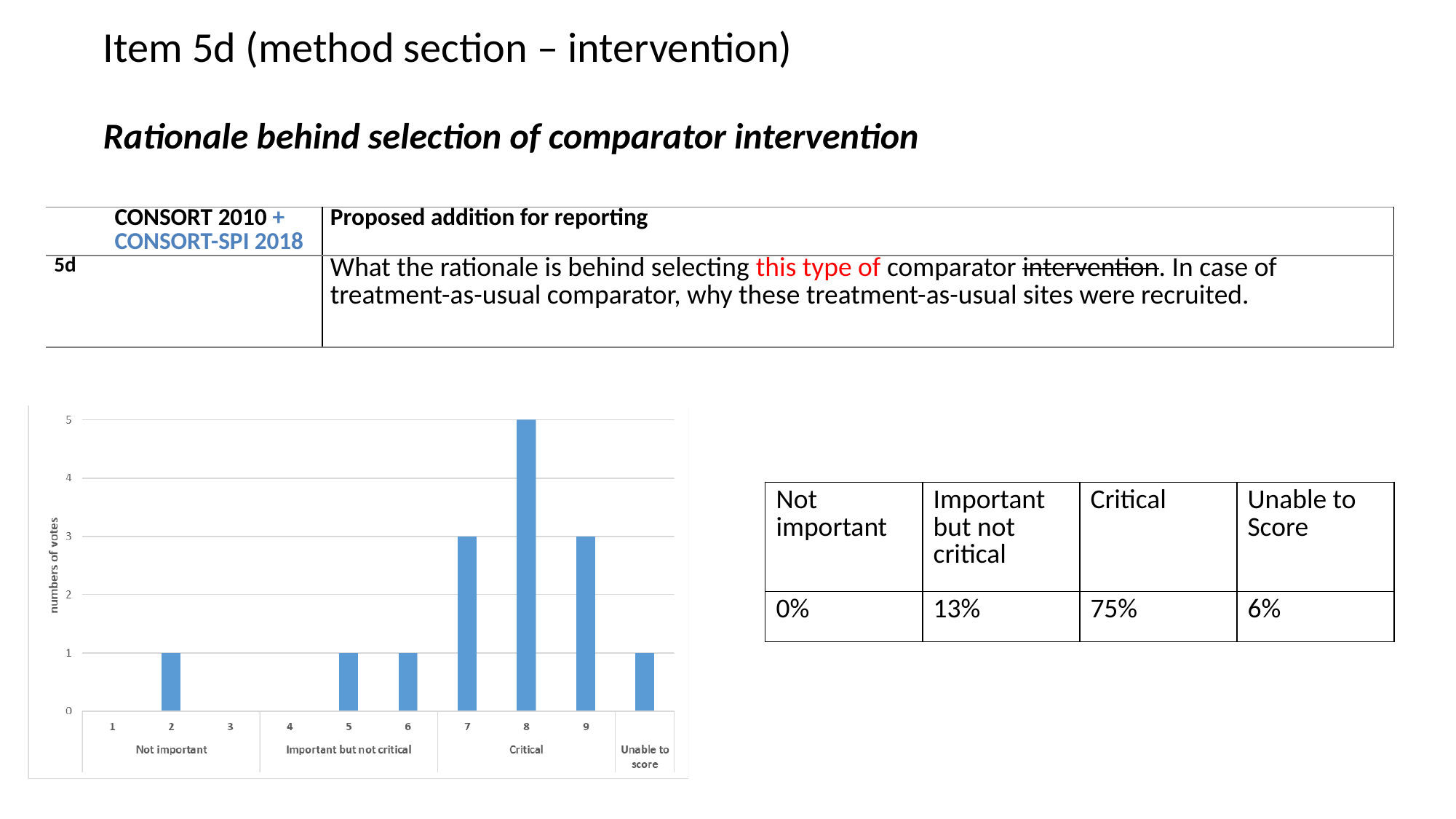

# Item 5d (method section – intervention) Rationale behind selection of comparator intervention
| | CONSORT 2010 + CONSORT-SPI 2018 | Proposed addition for reporting |
| --- | --- | --- |
| 5d | | What the rationale is behind selecting this type of comparator intervention. In case of treatment-as-usual comparator, why these treatment-as-usual sites were recruited. |
| Not important | Important but not critical | Critical | Unable to Score |
| --- | --- | --- | --- |
| 0% | 13% | 75% | 6% |

## Slide 10
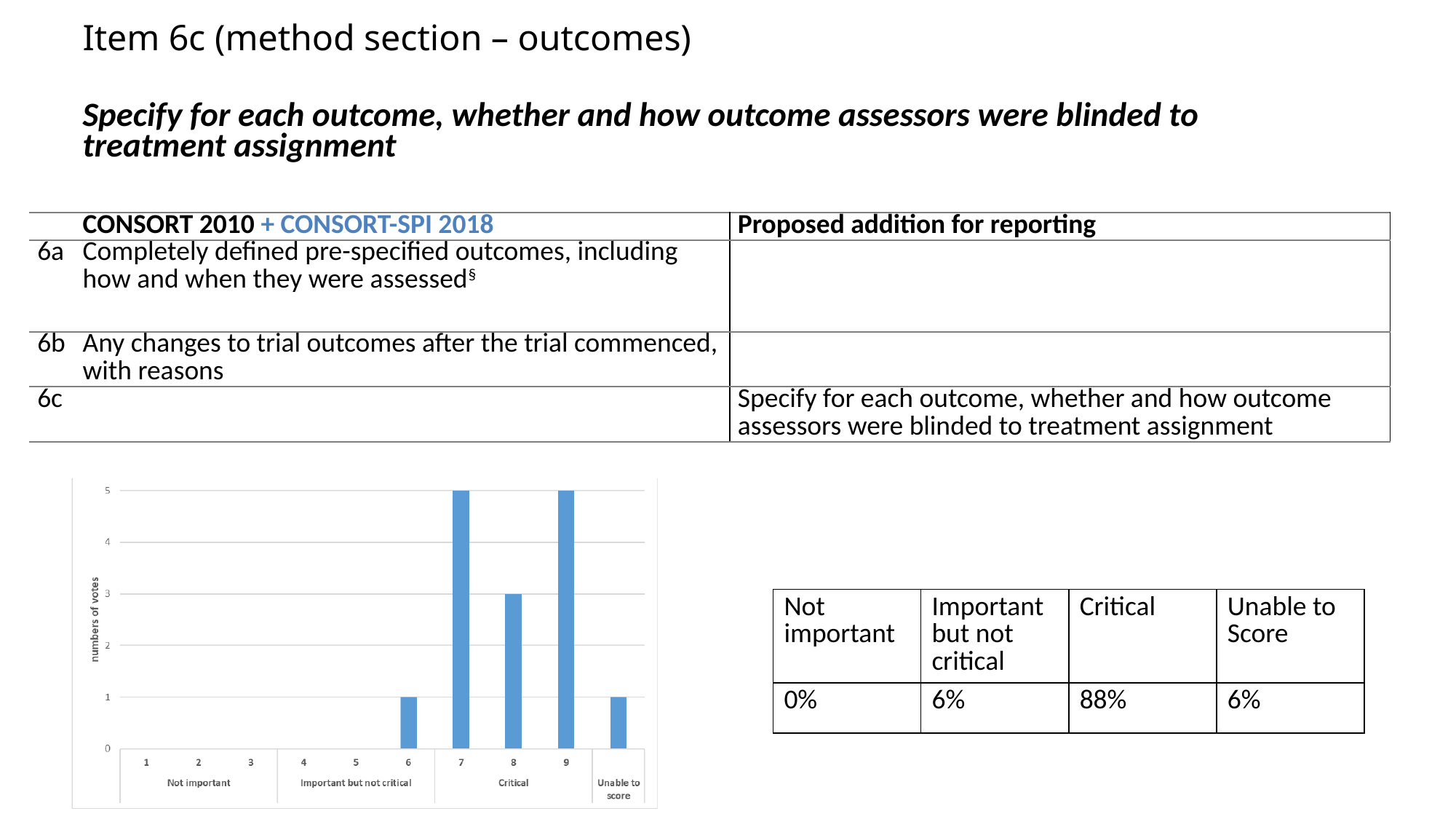

# Item 6c (method section – outcomes) Specify for each outcome, whether and how outcome assessors were blinded to treatment assignment
| | CONSORT 2010 + CONSORT-SPI 2018 | Proposed addition for reporting |
| --- | --- | --- |
| 6a | Completely defined pre-specified outcomes, including how and when they were assessed§ | |
| 6b | Any changes to trial outcomes after the trial commenced, with reasons | |
| 6c | | Specify for each outcome, whether and how outcome assessors were blinded to treatment assignment |
| Not important | Important but not critical | Critical | Unable to Score |
| --- | --- | --- | --- |
| 0% | 6% | 88% | 6% |

## Slide 11
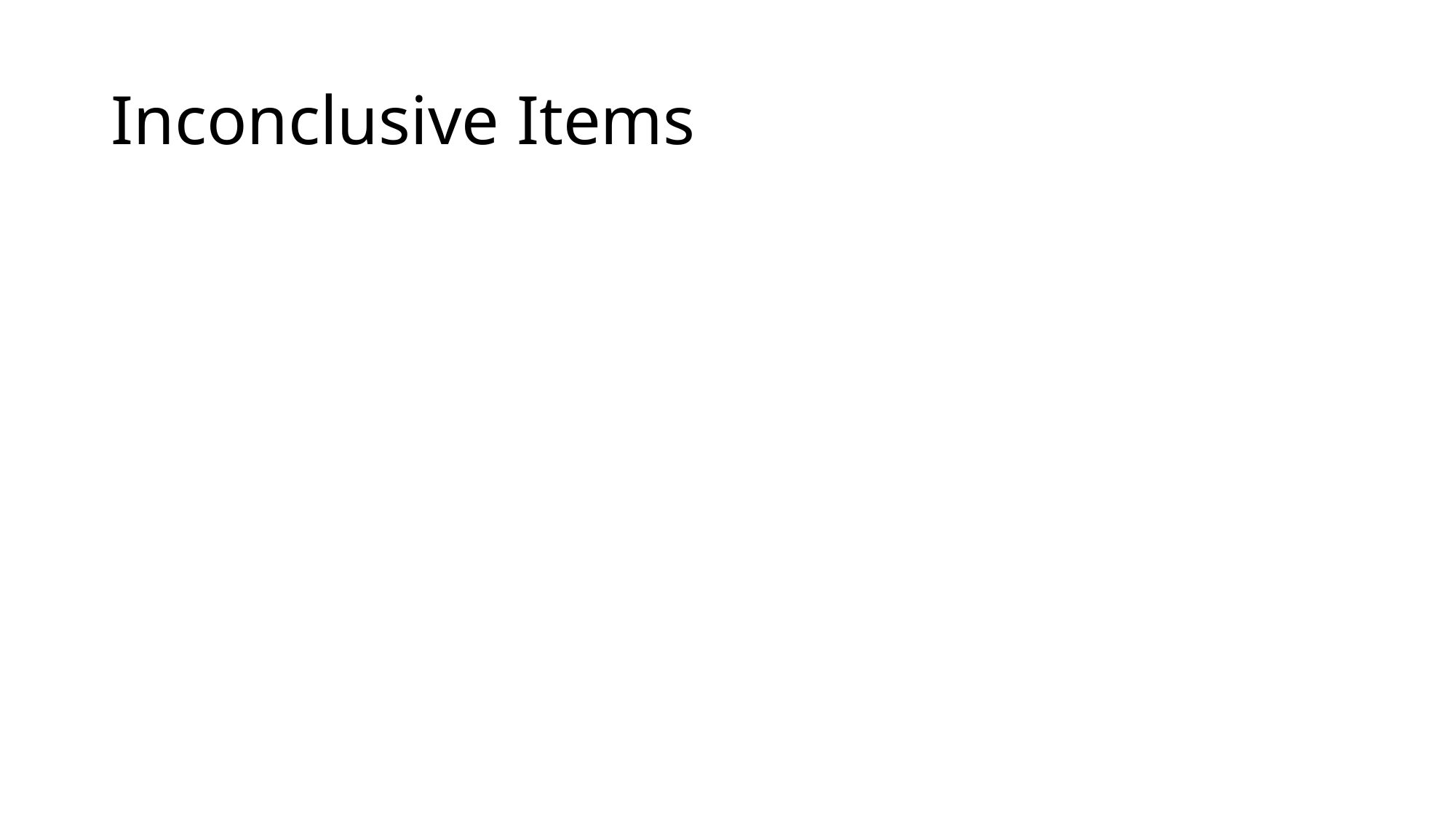

# Inconclusive Items

## Slide 12
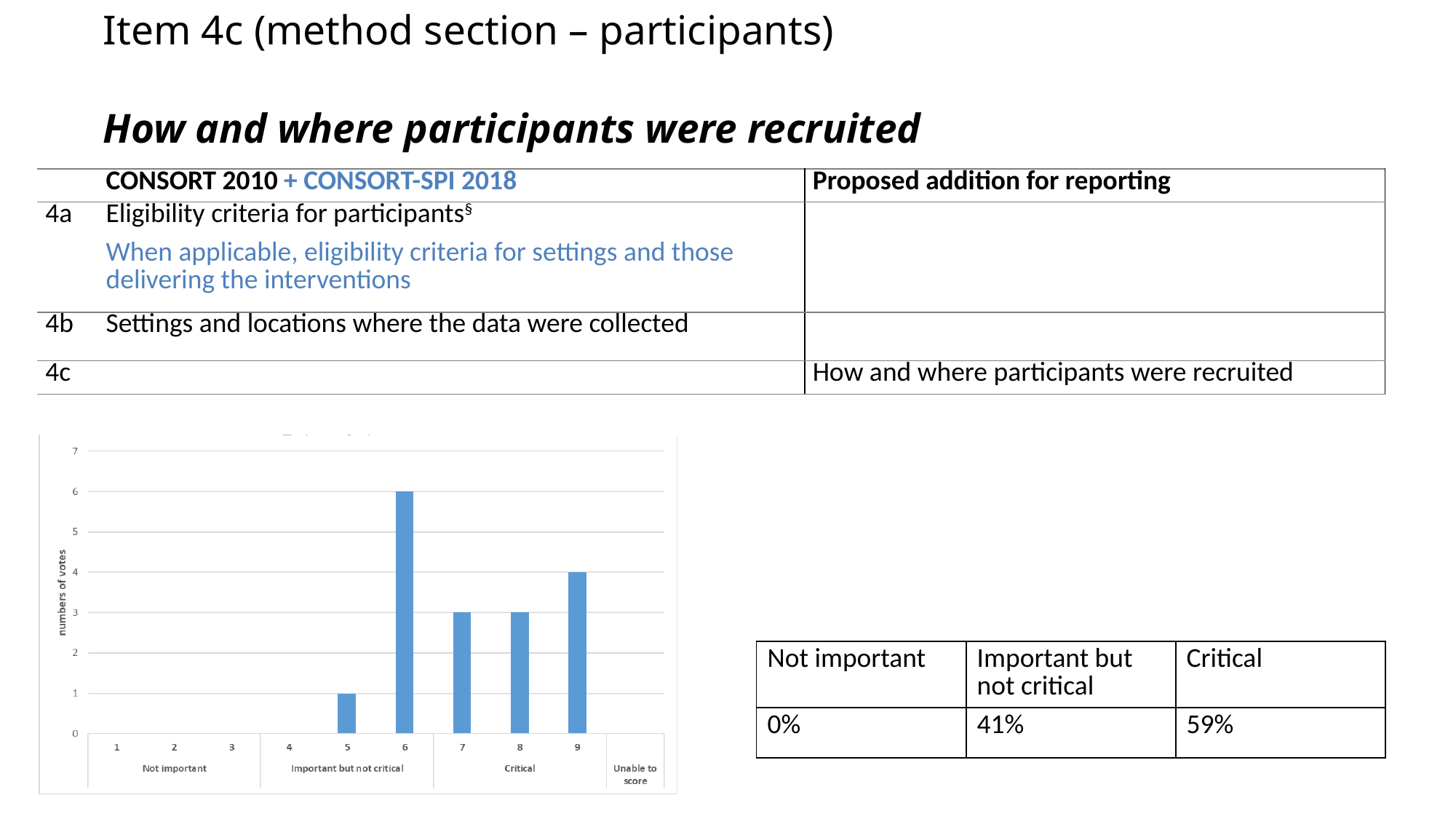

# Item 4c (method section – participants) How and where participants were recruited
| | CONSORT 2010 + CONSORT-SPI 2018 | Proposed addition for reporting |
| --- | --- | --- |
| 4a | Eligibility criteria for participants§ When applicable, eligibility criteria for settings and those delivering the interventions | |
| 4b | Settings and locations where the data were collected | |
| 4c | | How and where participants were recruited |
| Not important | Important but not critical | Critical |
| --- | --- | --- |
| 0% | 41% | 59% |

## Slide 13
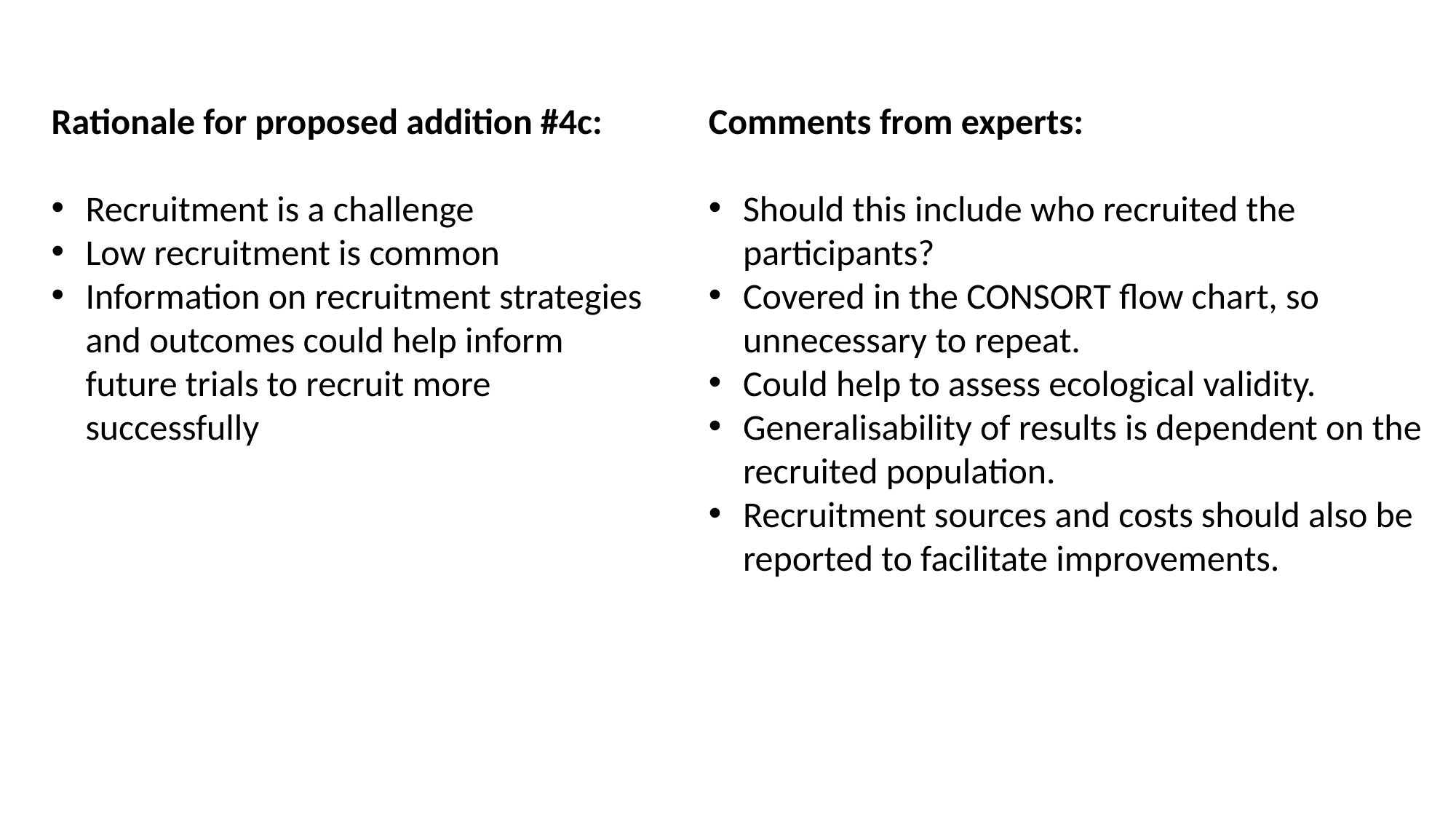

Rationale for proposed addition #4c:
Recruitment is a challenge
Low recruitment is common
Information on recruitment strategies and outcomes could help inform future trials to recruit more successfully
Comments from experts:
Should this include who recruited the participants?
Covered in the CONSORT flow chart, so unnecessary to repeat.
Could help to assess ecological validity.
Generalisability of results is dependent on the recruited population.
Recruitment sources and costs should also be reported to facilitate improvements.

## Slide 14
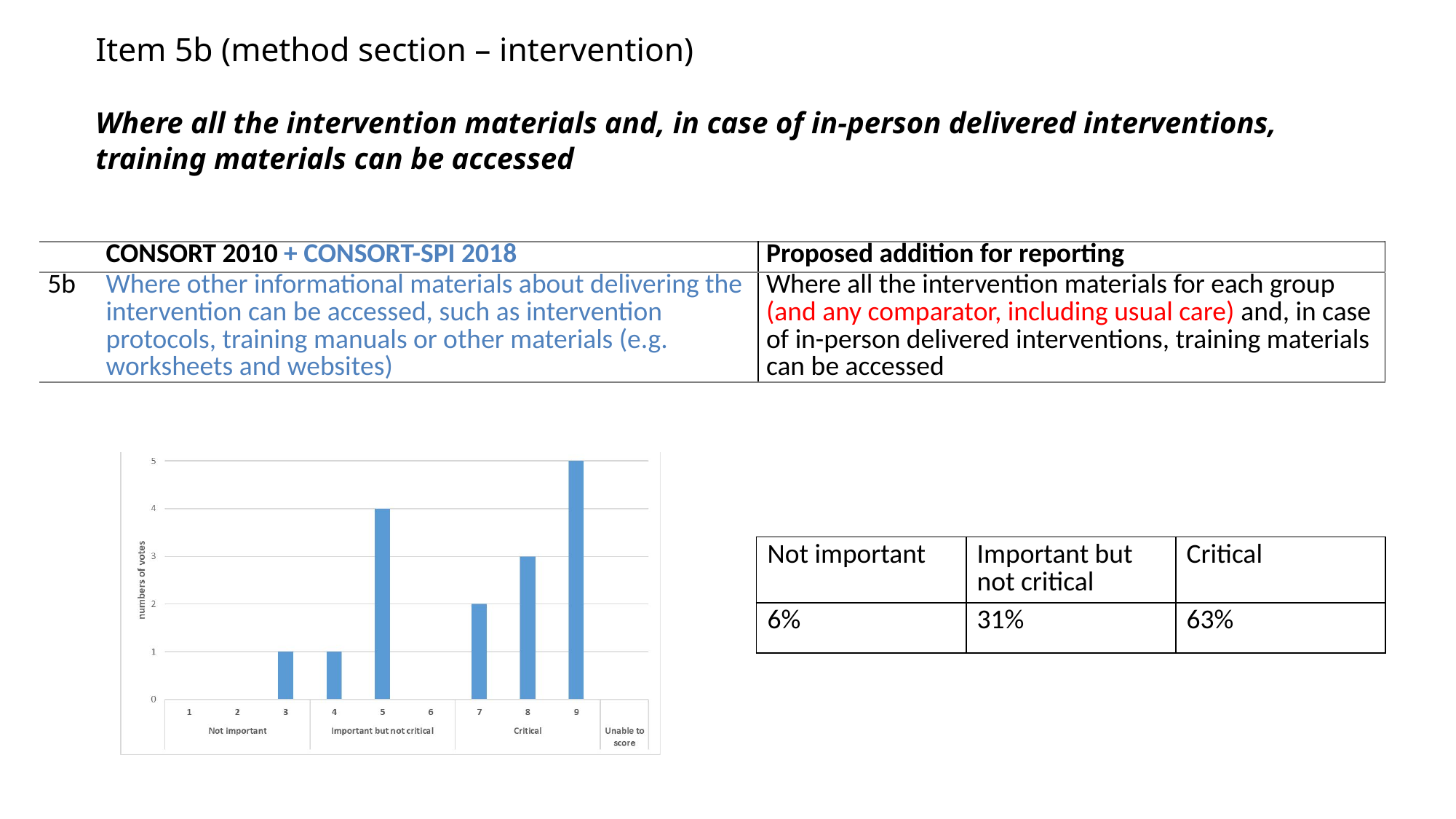

# Item 5b (method section – intervention) Where all the intervention materials and, in case of in-person delivered interventions, training materials can be accessed
| | CONSORT 2010 + CONSORT-SPI 2018 | Proposed addition for reporting |
| --- | --- | --- |
| 5b | Where other informational materials about delivering the intervention can be accessed, such as intervention protocols, training manuals or other materials (e.g. worksheets and websites) | Where all the intervention materials for each group (and any comparator, including usual care) and, in case of in-person delivered interventions, training materials can be accessed |
| Not important | Important but not critical | Critical |
| --- | --- | --- |
| 6% | 31% | 63% |

## Slide 15
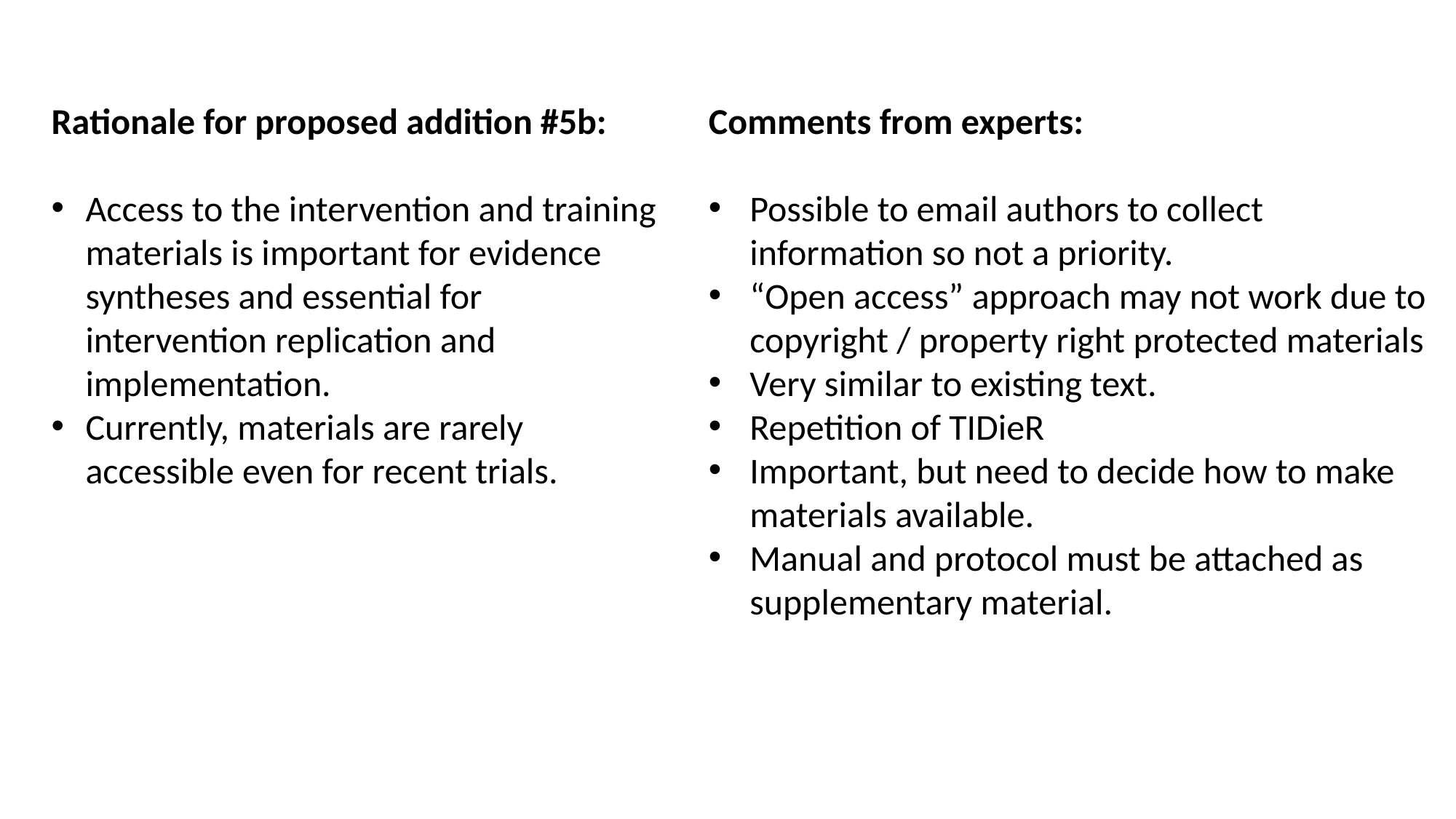

Rationale for proposed addition #5b:
Access to the intervention and training materials is important for evidence syntheses and essential for intervention replication and implementation.
Currently, materials are rarely accessible even for recent trials.
Comments from experts:
Possible to email authors to collect information so not a priority.
“Open access” approach may not work due to copyright / property right protected materials
Very similar to existing text.
Repetition of TIDieR
Important, but need to decide how to make materials available.
Manual and protocol must be attached as supplementary material.

## Slide 16
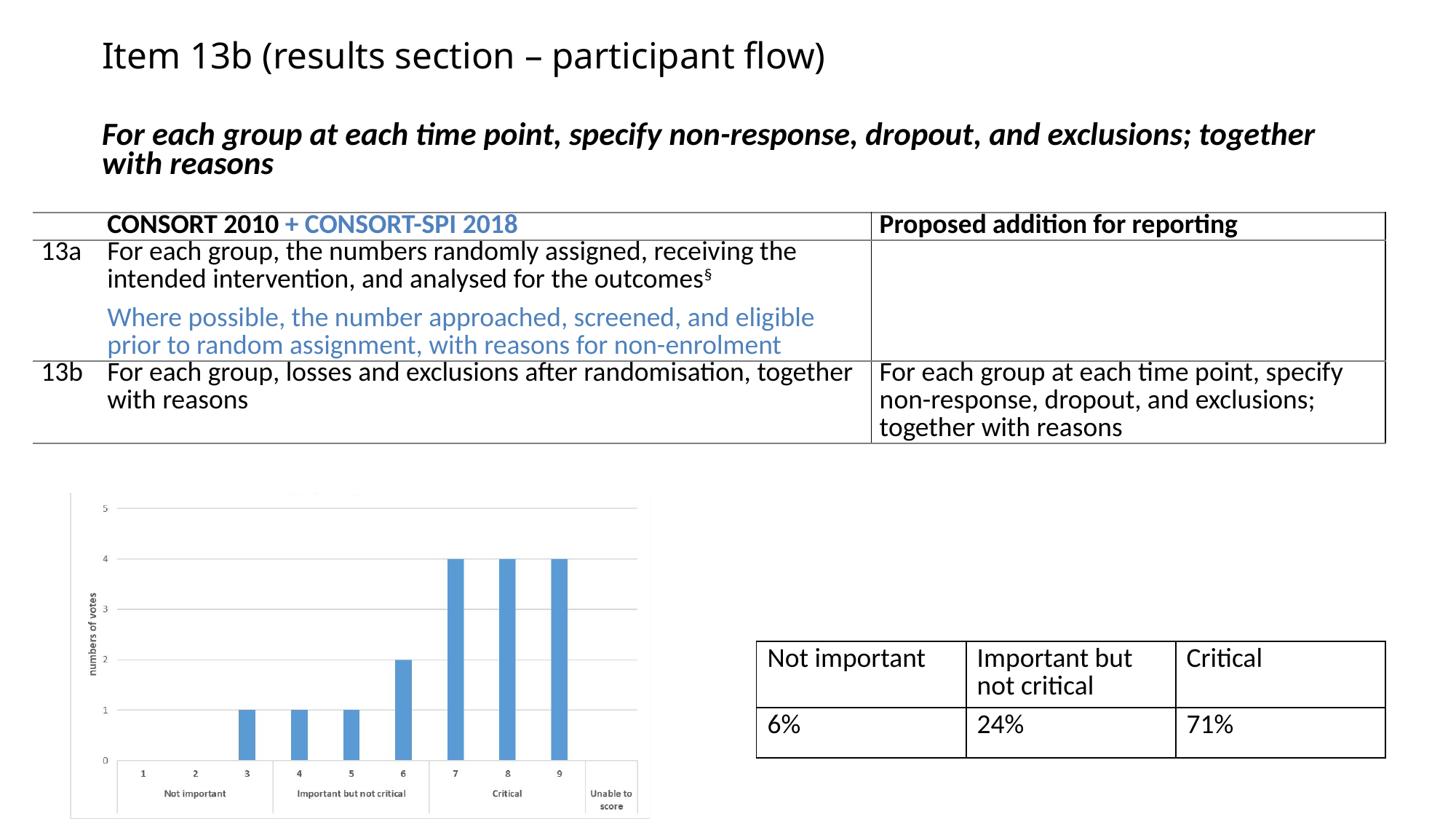

# Item 13b (results section – participant flow) For each group at each time point, specify non-response, dropout, and exclusions; together with reasons
| | CONSORT 2010 + CONSORT-SPI 2018 | Proposed addition for reporting |
| --- | --- | --- |
| 13a | For each group, the numbers randomly assigned, receiving the intended intervention, and analysed for the outcomes§ Where possible, the number approached, screened, and eligible prior to random assignment, with reasons for non-enrolment | |
| 13b | For each group, losses and exclusions after randomisation, together with reasons | For each group at each time point, specify non-response, dropout, and exclusions; together with reasons |
| Not important | Important but not critical | Critical |
| --- | --- | --- |
| 6% | 24% | 71% |

## Slide 17
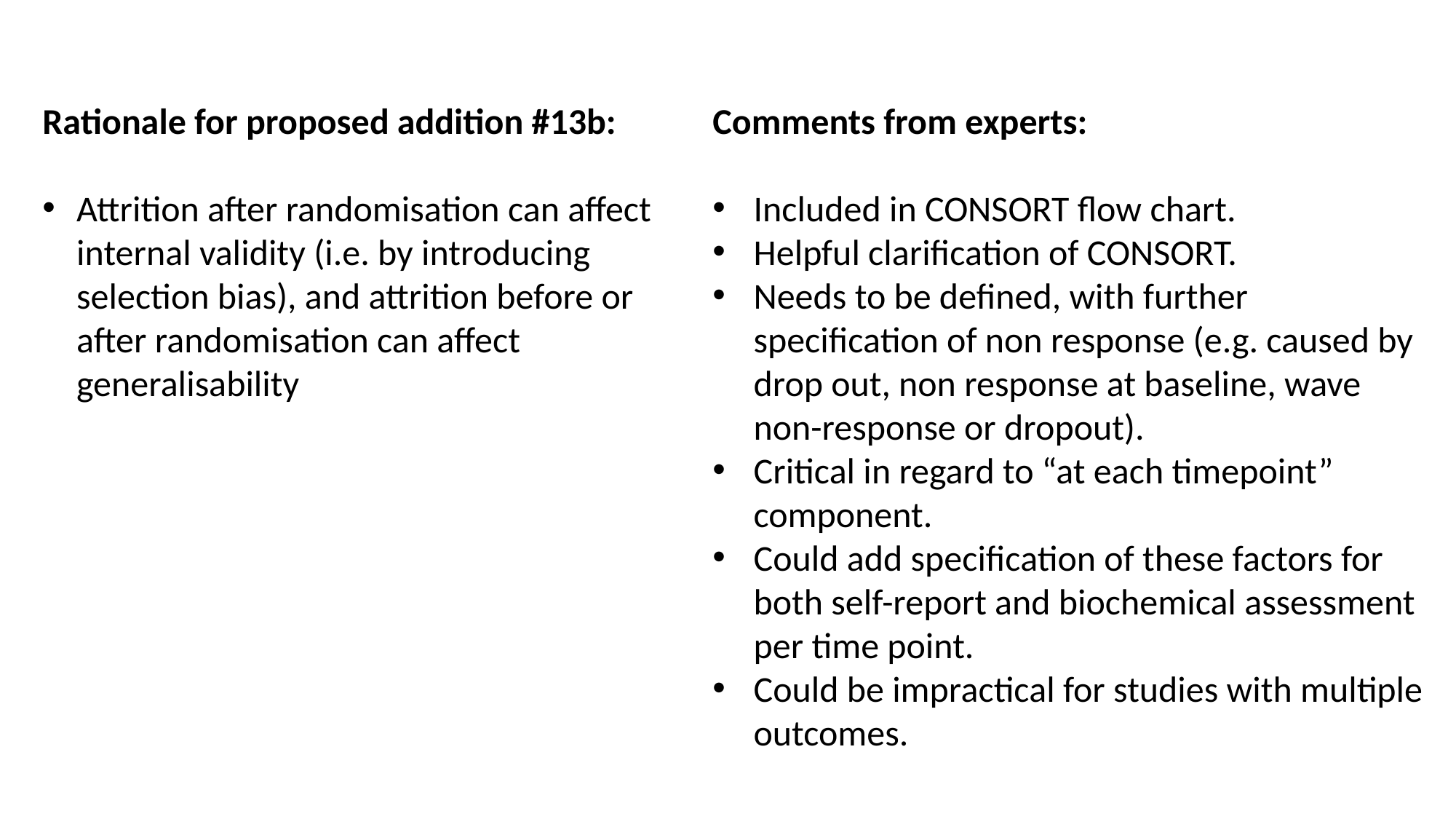

Rationale for proposed addition #13b:
Attrition after randomisation can affect internal validity (i.e. by introducing selection bias), and attrition before or after randomisation can affect generalisability
Comments from experts:
Included in CONSORT flow chart.
Helpful clarification of CONSORT.
Needs to be defined, with further specification of non response (e.g. caused by drop out, non response at baseline, wave non-response or dropout).
Critical in regard to “at each timepoint” component.
Could add specification of these factors for both self-report and biochemical assessment per time point.
Could be impractical for studies with multiple outcomes.

## Slide 18
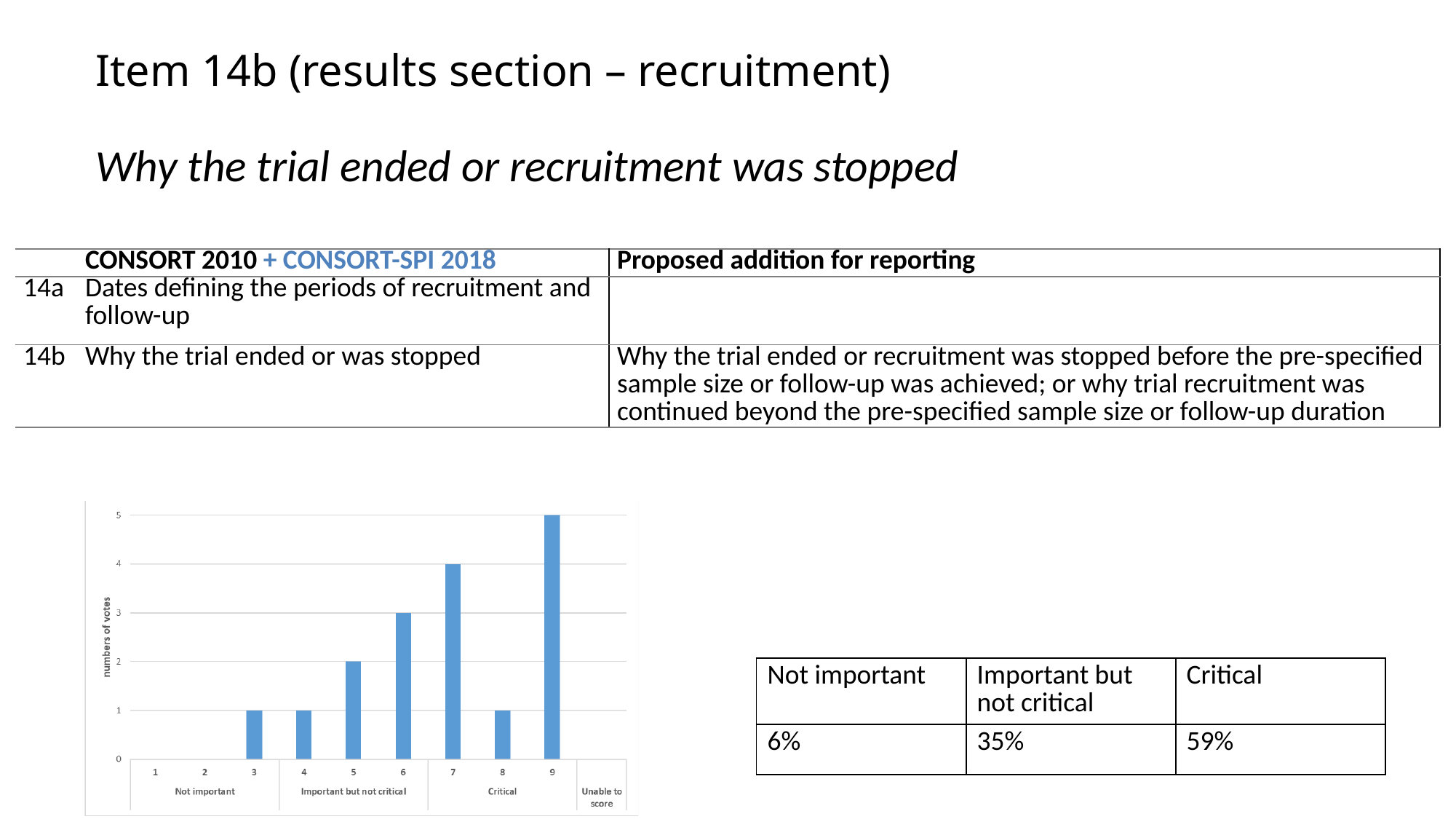

# Item 14b (results section – recruitment) Why the trial ended or recruitment was stopped
| | CONSORT 2010 + CONSORT-SPI 2018 | Proposed addition for reporting |
| --- | --- | --- |
| 14a | Dates defining the periods of recruitment and follow-up | |
| 14b | Why the trial ended or was stopped | Why the trial ended or recruitment was stopped before the pre-specified sample size or follow-up was achieved; or why trial recruitment was continued beyond the pre-specified sample size or follow-up duration |
| Not important | Important but not critical | Critical |
| --- | --- | --- |
| 6% | 35% | 59% |

## Slide 19
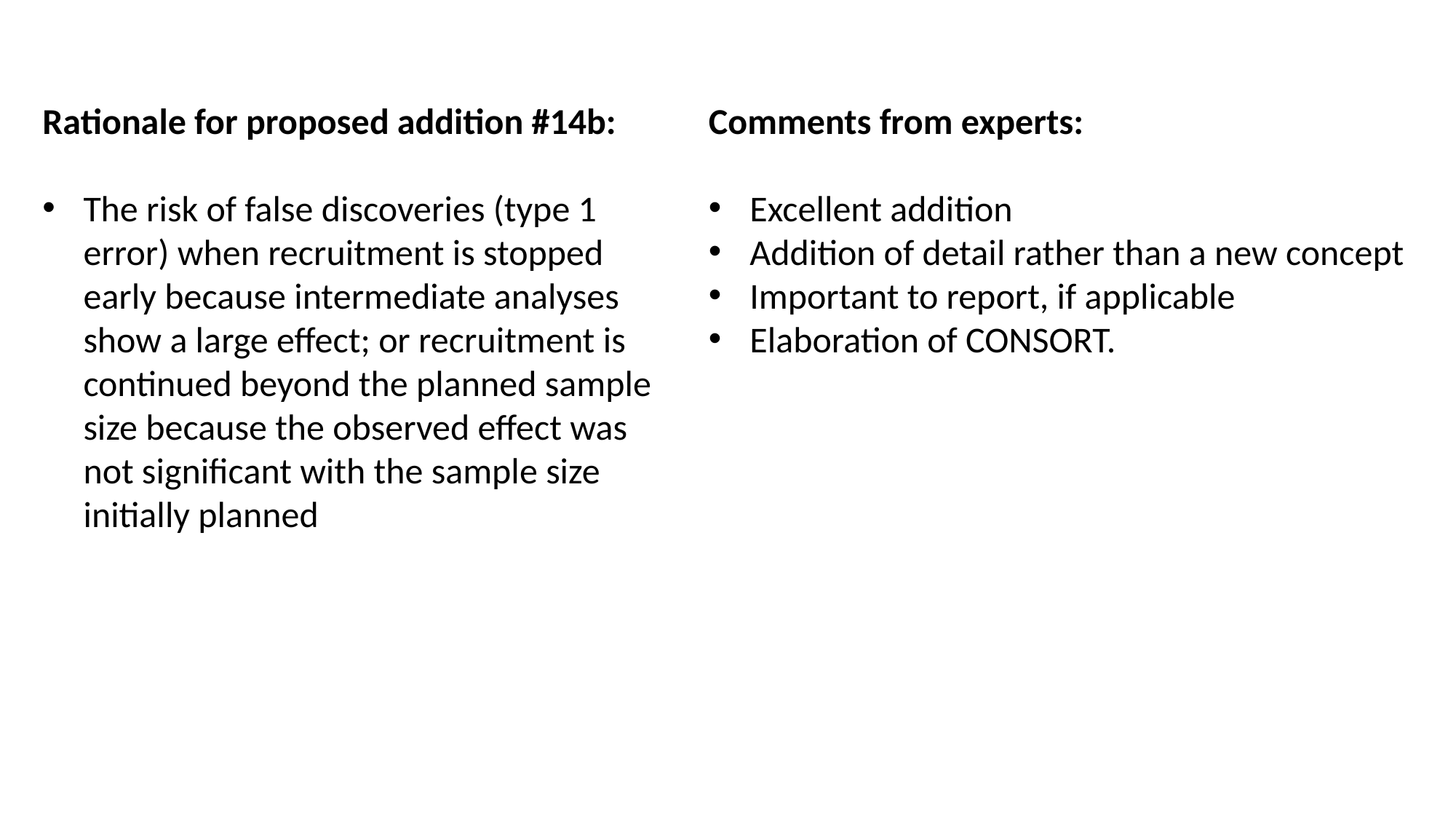

Rationale for proposed addition #14b:
The risk of false discoveries (type 1 error) when recruitment is stopped early because intermediate analyses show a large effect; or recruitment is continued beyond the planned sample size because the observed effect was not significant with the sample size initially planned
Comments from experts:
Excellent addition
Addition of detail rather than a new concept
Important to report, if applicable
Elaboration of CONSORT.

## Slide 20
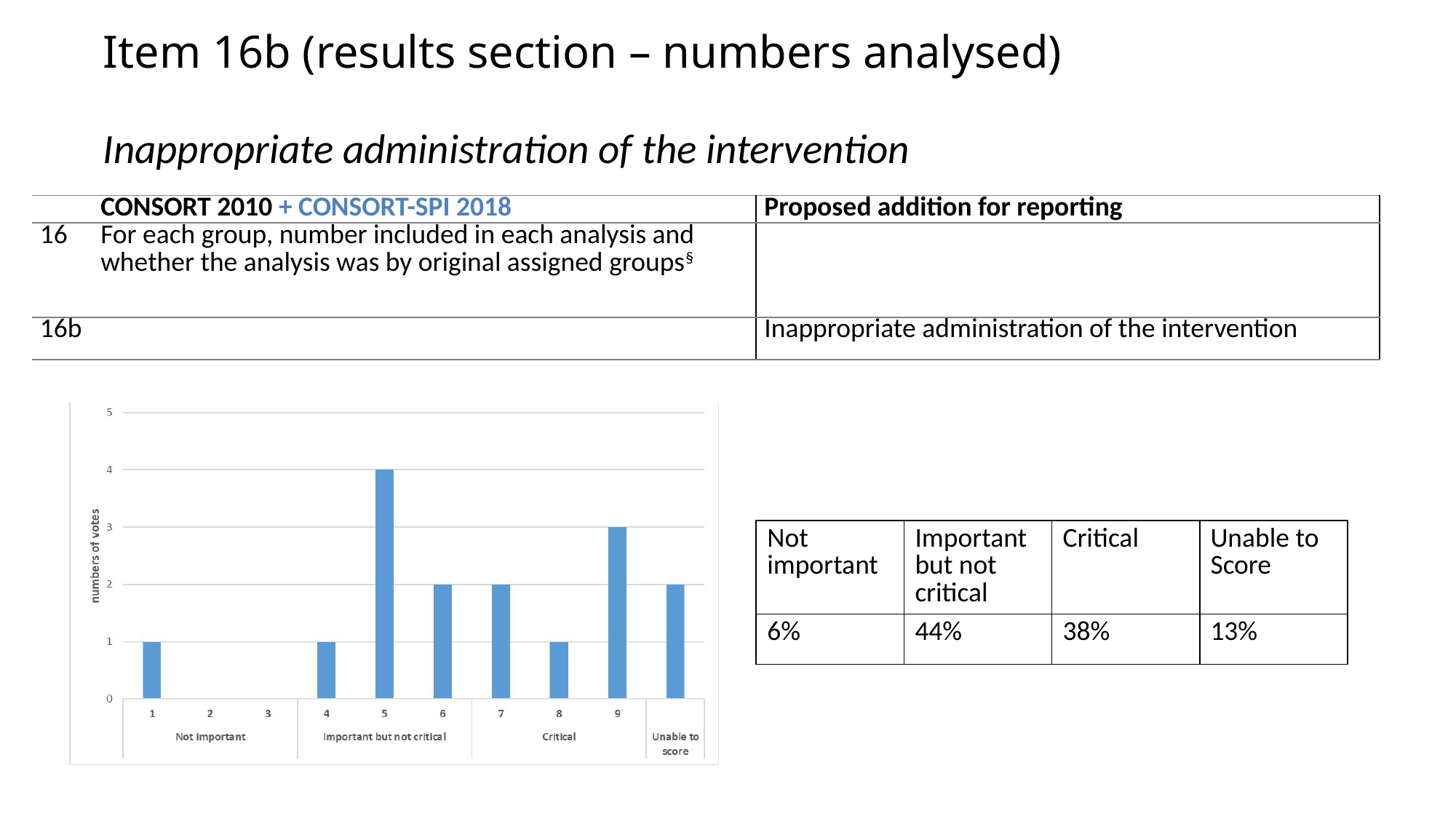

# Item 16b (results section – numbers analysed) Inappropriate administration of the intervention
| | CONSORT 2010 + CONSORT-SPI 2018 | Proposed addition for reporting |
| --- | --- | --- |
| 16 | For each group, number included in each analysis and whether the analysis was by original assigned groups§ | |
| 16b | | Inappropriate administration of the intervention |
| Not important | Important but not critical | Critical | Unable to Score |
| --- | --- | --- | --- |
| 6% | 44% | 38% | 13% |

## Slide 21
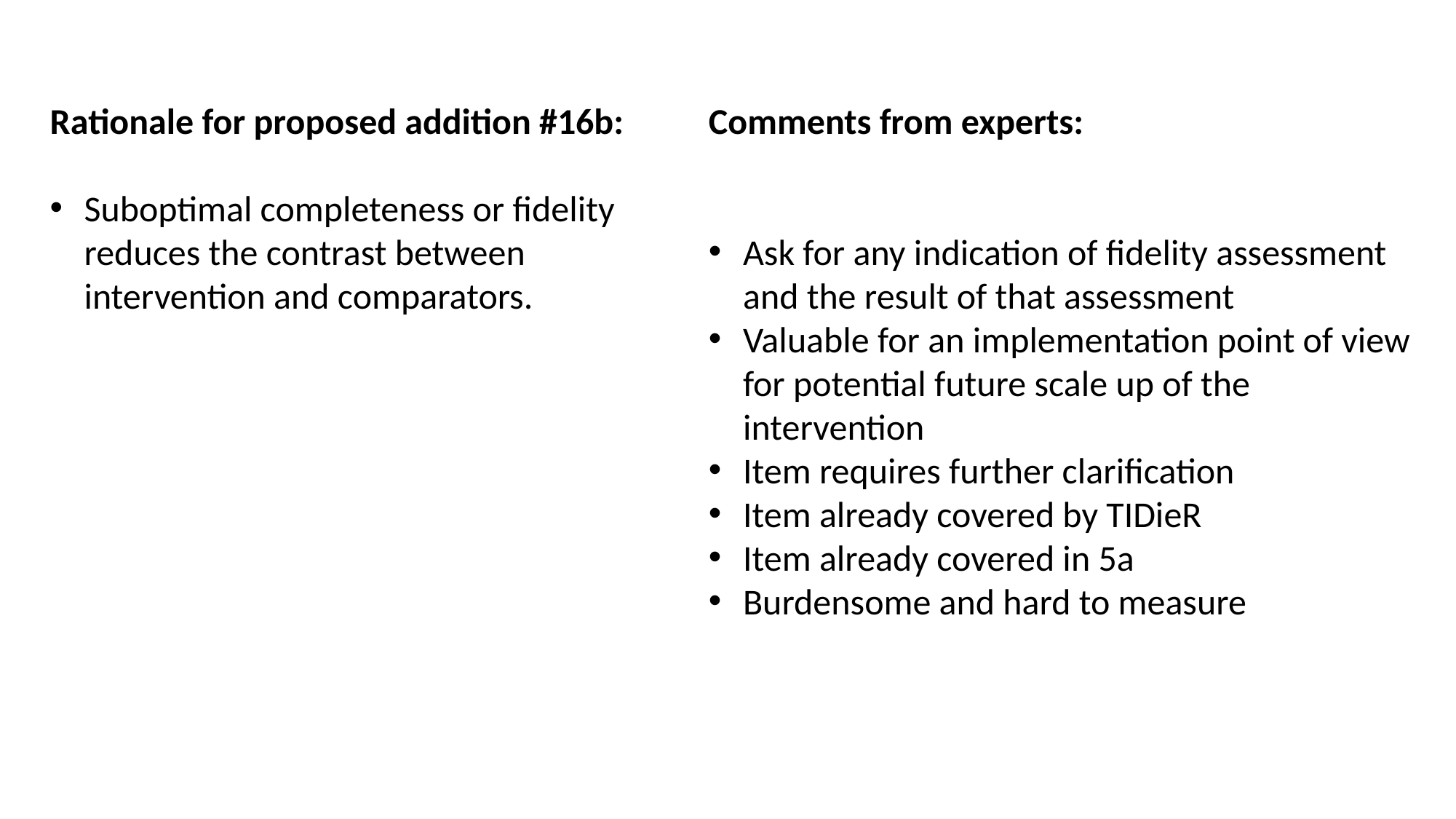

Rationale for proposed addition #16b:
Suboptimal completeness or fidelity reduces the contrast between intervention and comparators.
Comments from experts:
Ask for any indication of fidelity assessment and the result of that assessment
Valuable for an implementation point of view for potential future scale up of the intervention
Item requires further clarification
Item already covered by TIDieR
Item already covered in 5a
Burdensome and hard to measure

## Slide 22
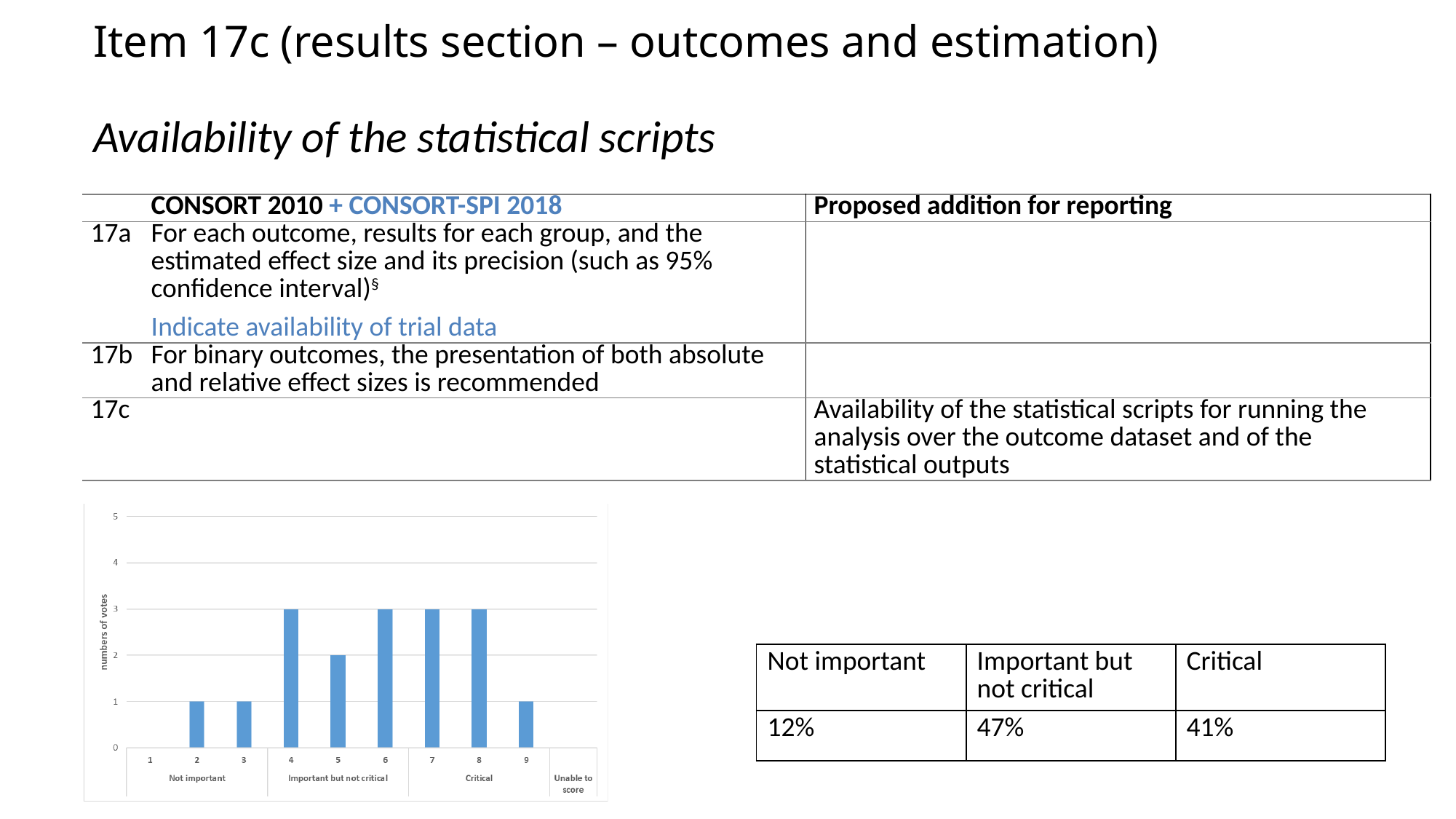

# Item 17c (results section – outcomes and estimation) Availability of the statistical scripts
| | CONSORT 2010 + CONSORT-SPI 2018 | Proposed addition for reporting |
| --- | --- | --- |
| 17a | For each outcome, results for each group, and the estimated effect size and its precision (such as 95% confidence interval)§ Indicate availability of trial data | |
| 17b | For binary outcomes, the presentation of both absolute and relative effect sizes is recommended | |
| 17c | | Availability of the statistical scripts for running the analysis over the outcome dataset and of the statistical outputs |
| Not important | Important but not critical | Critical |
| --- | --- | --- |
| 12% | 47% | 41% |

## Slide 23
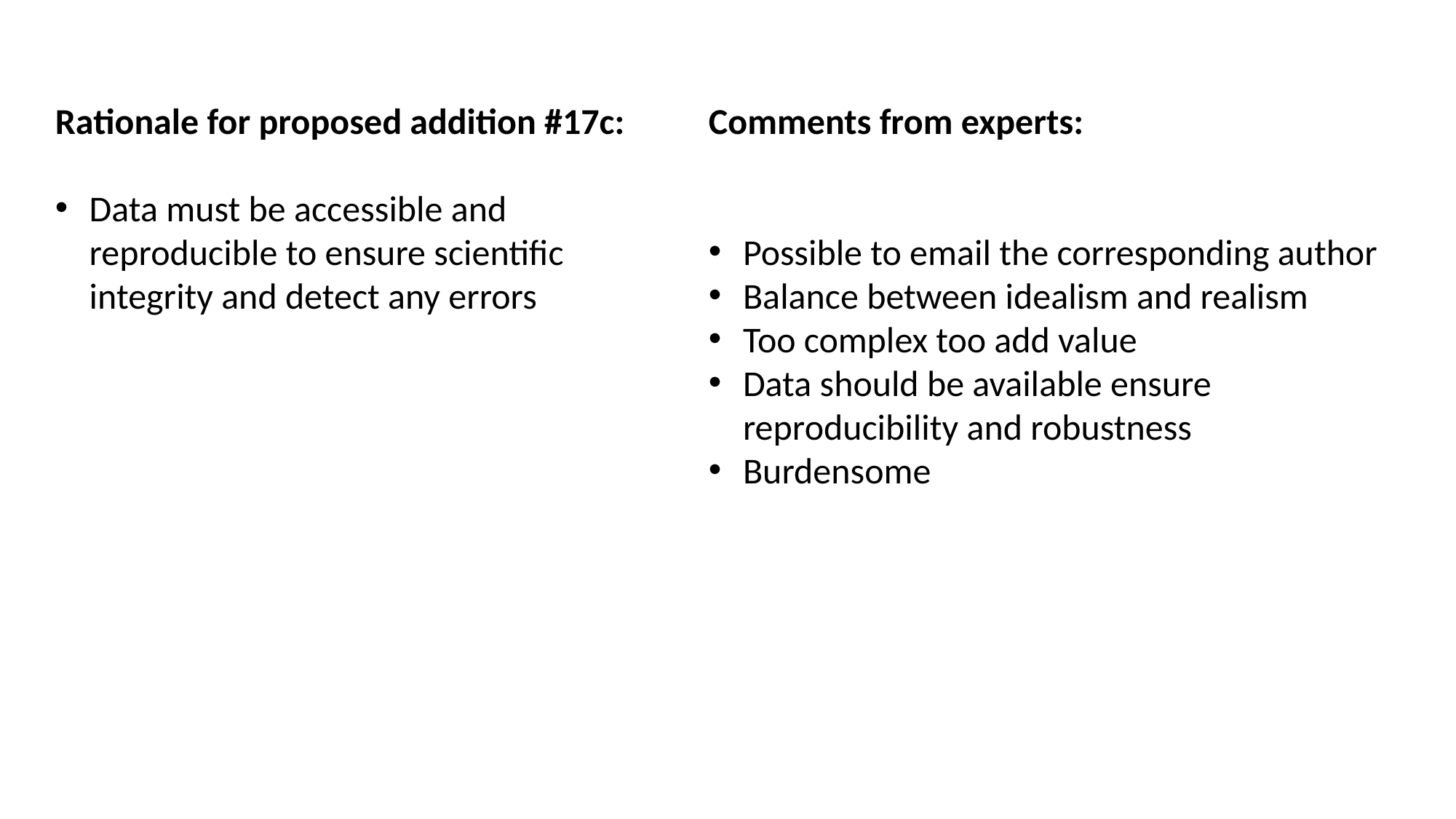

Rationale for proposed addition #17c:
Data must be accessible and reproducible to ensure scientific integrity and detect any errors
Comments from experts:
Possible to email the corresponding author
Balance between idealism and realism
Too complex too add value
Data should be available ensure reproducibility and robustness
Burdensome

## Slide 24
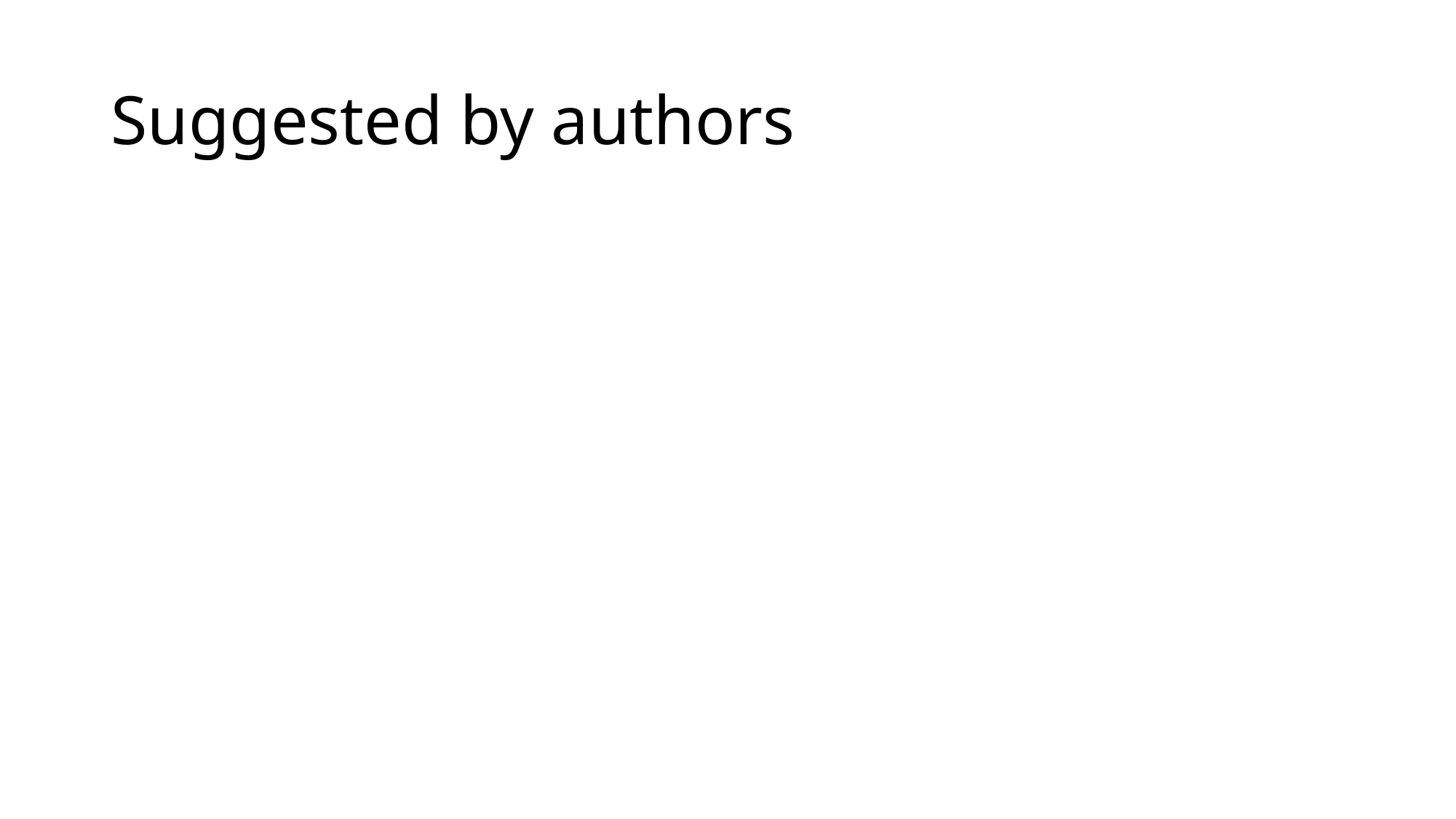

# Suggested by authors

## Slide 25
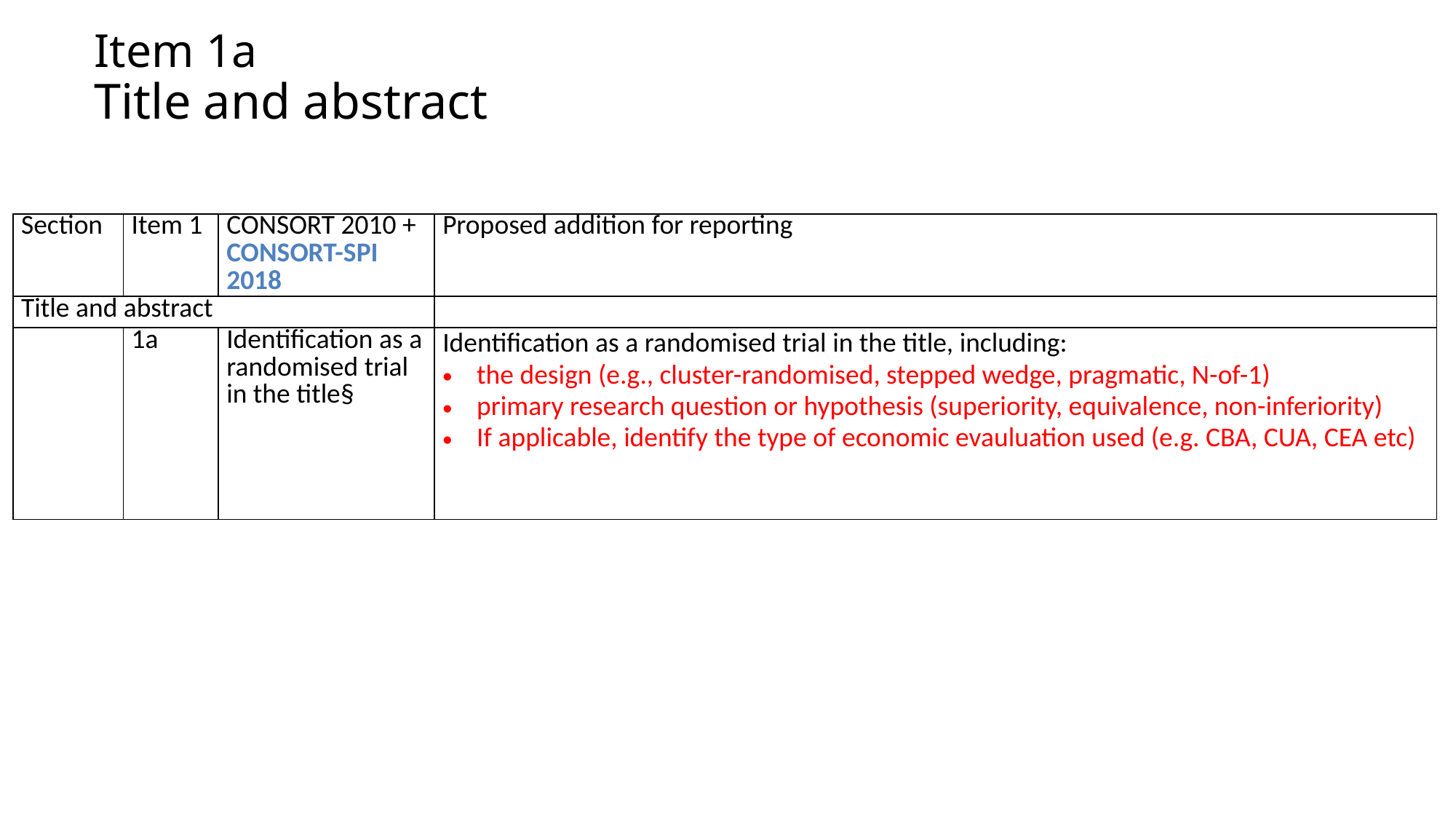

# Item 1a Title and abstract
| Section | Item 1 | CONSORT 2010 + CONSORT-SPI 2018 | Proposed addition for reporting |
| --- | --- | --- | --- |
| Title and abstract | | | |
| | 1a | Identification as a randomised trial in the title§ | Identification as a randomised trial in the title, including: the design (e.g., cluster-randomised, stepped wedge, pragmatic, N-of-1) primary research question or hypothesis (superiority, equivalence, non-inferiority) If applicable, identify the type of economic evauluation used (e.g. CBA, CUA, CEA etc) |

## Slide 26
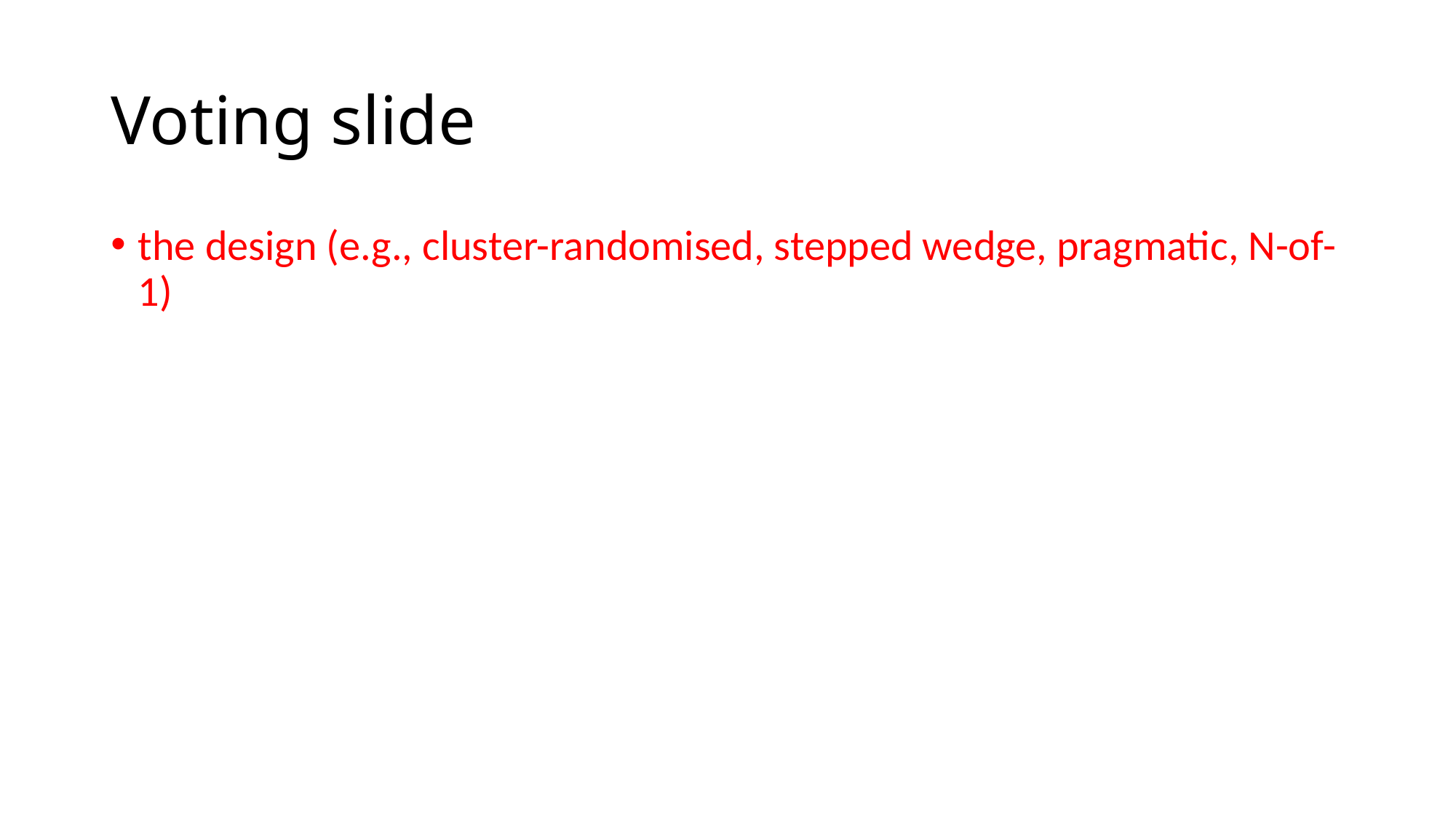

# Voting slide
the design (e.g., cluster-randomised, stepped wedge, pragmatic, N-of-1)

## Slide 27
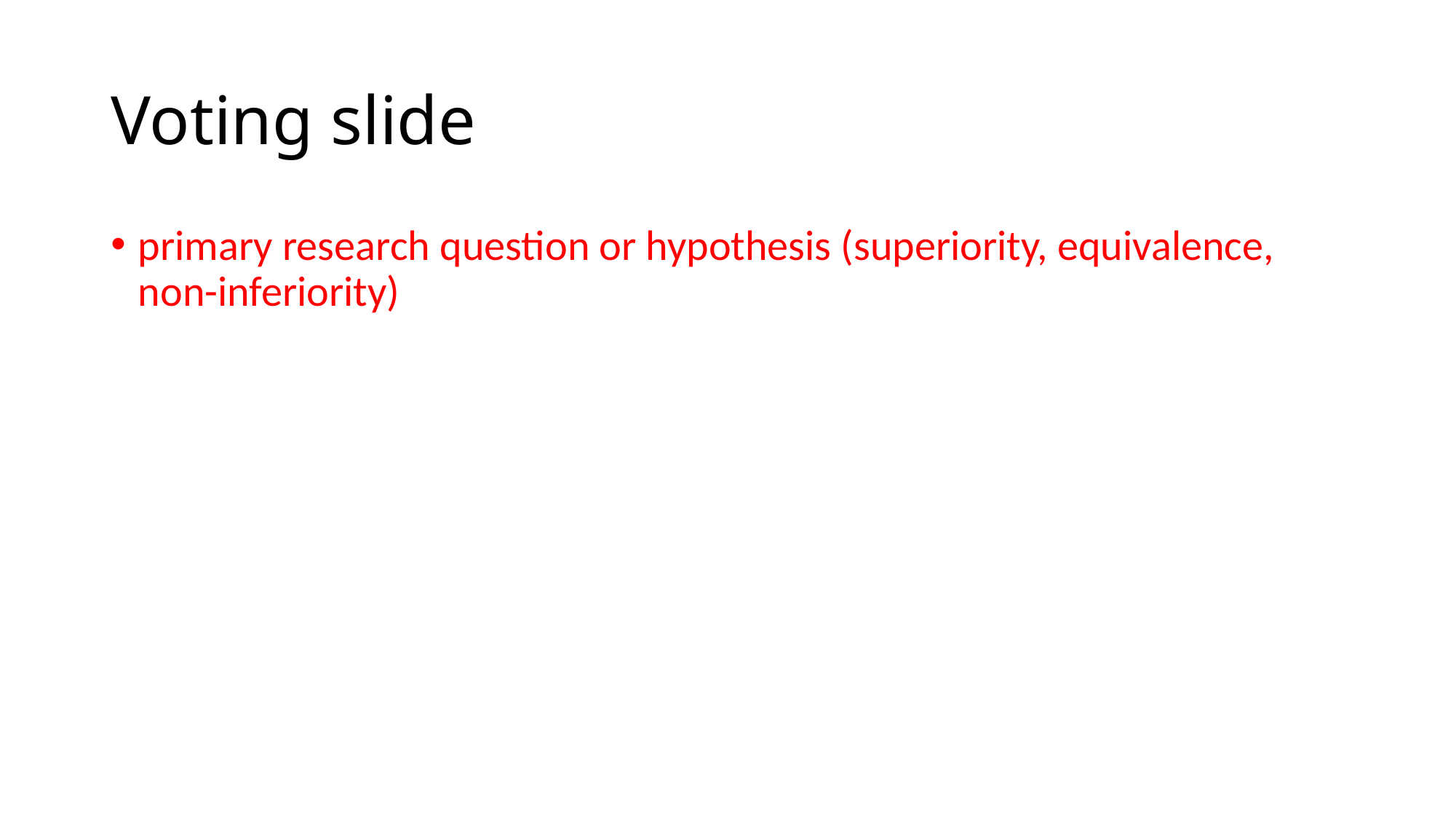

# Voting slide
primary research question or hypothesis (superiority, equivalence, non-inferiority)

## Slide 28
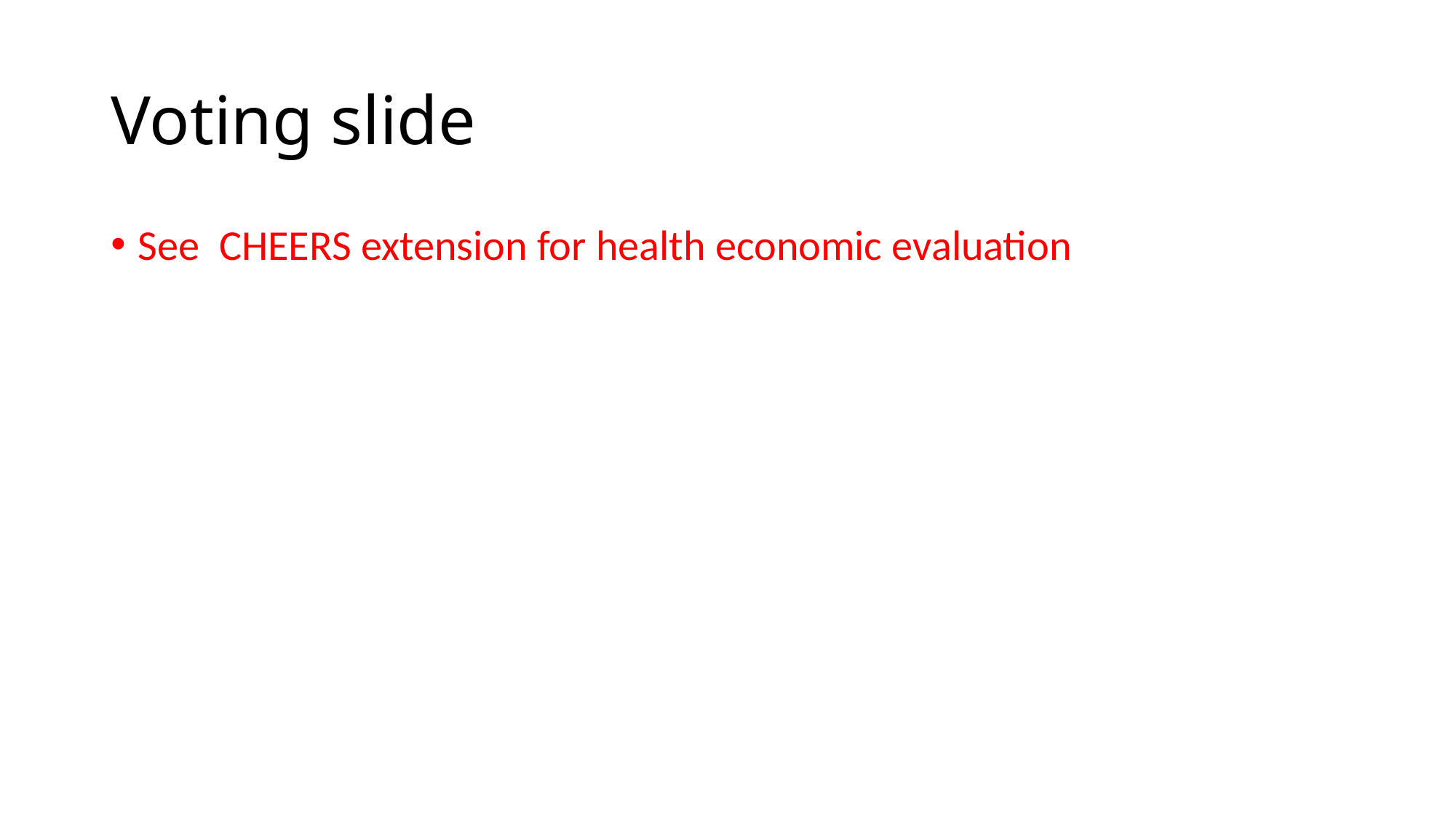

# Voting slide
See CHEERS extension for health economic evaluation

## Slide 29
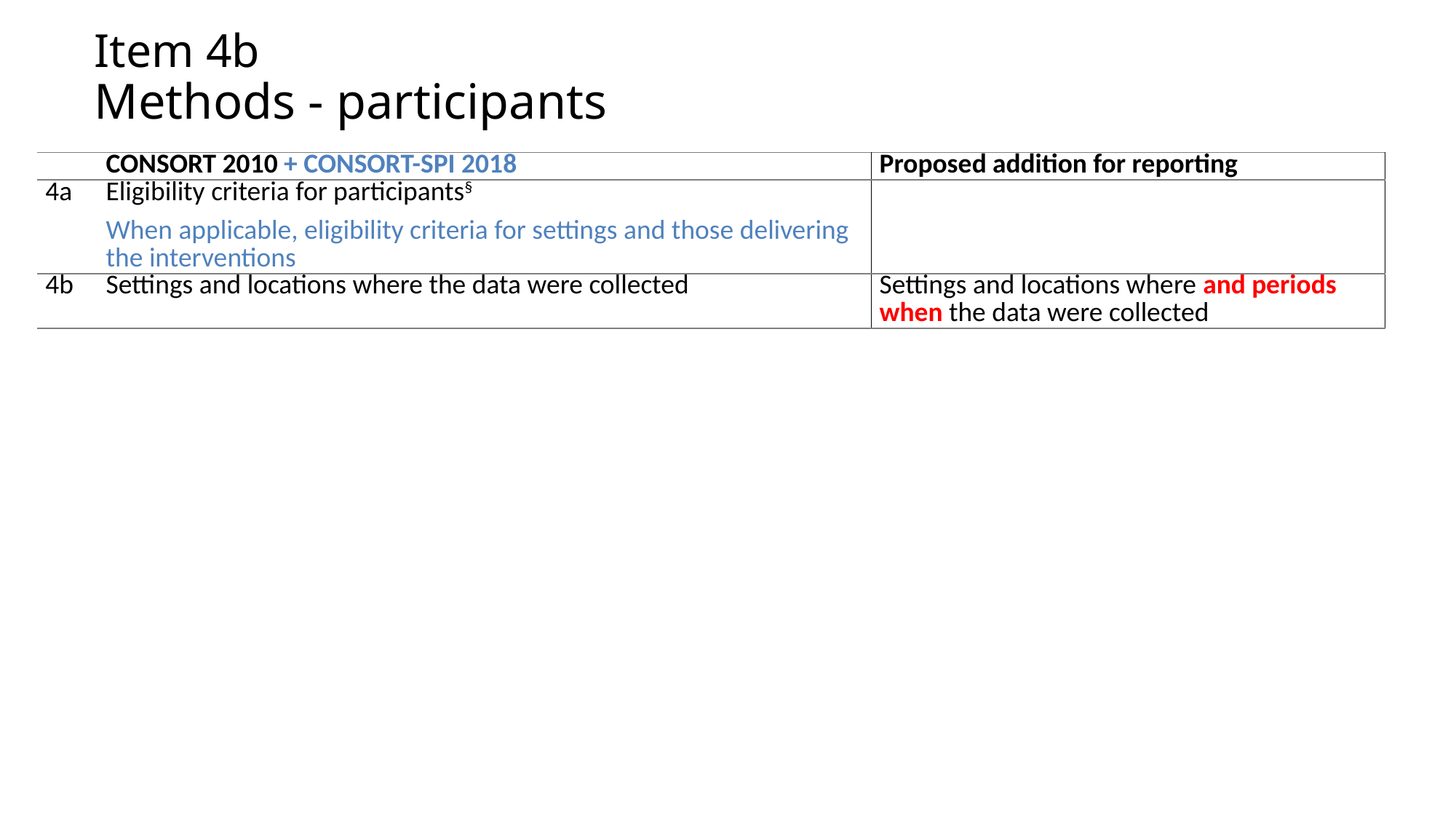

# Item 4b Methods - participants
| | CONSORT 2010 + CONSORT-SPI 2018 | Proposed addition for reporting |
| --- | --- | --- |
| 4a | Eligibility criteria for participants§ When applicable, eligibility criteria for settings and those delivering the interventions | |
| 4b | Settings and locations where the data were collected | Settings and locations where and periods when the data were collected |

## Slide 30
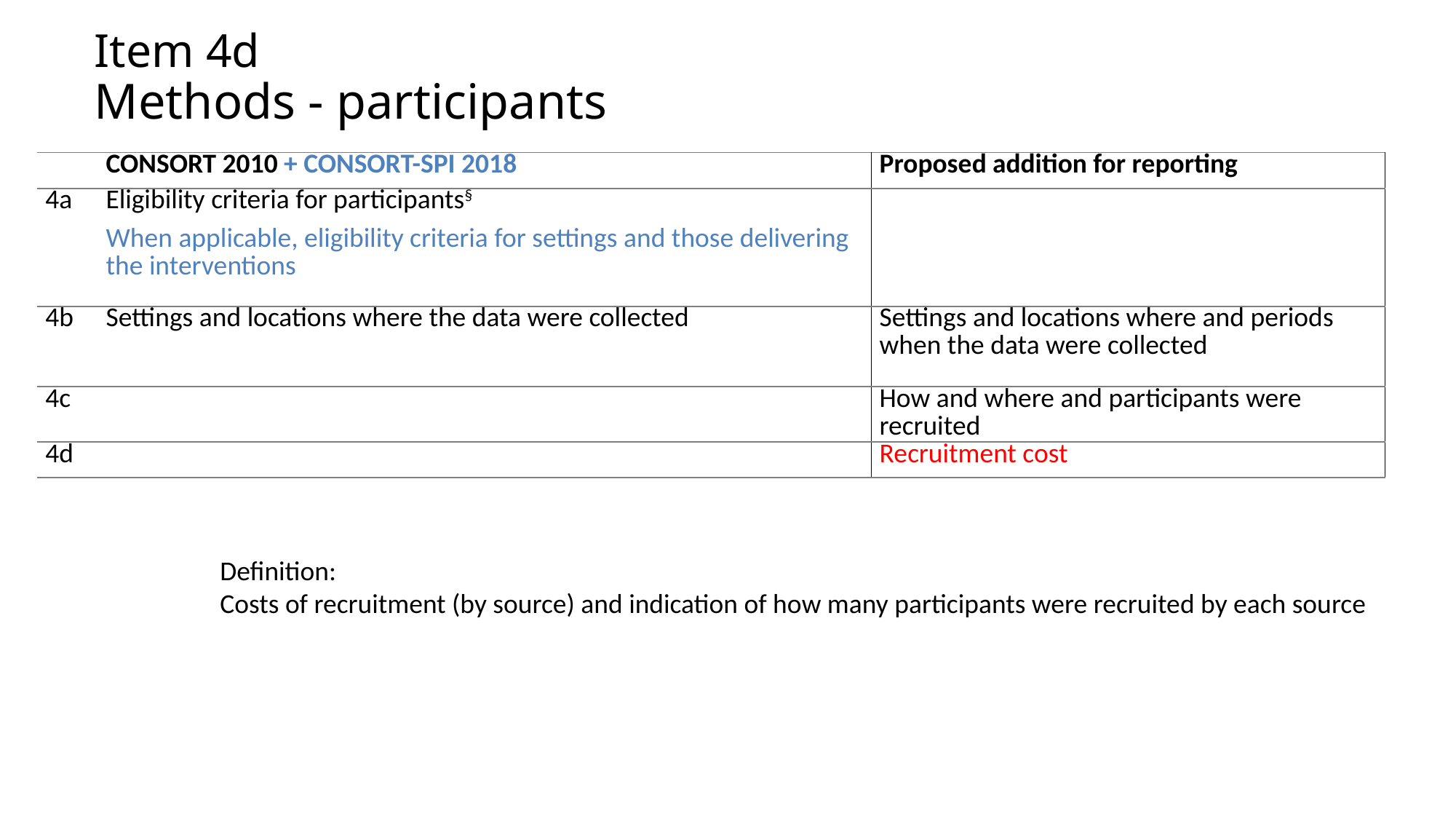

# Item 4d Methods - participants
| | CONSORT 2010 + CONSORT-SPI 2018 | Proposed addition for reporting |
| --- | --- | --- |
| 4a | Eligibility criteria for participants§ When applicable, eligibility criteria for settings and those delivering the interventions | |
| 4b | Settings and locations where the data were collected | Settings and locations where and periods when the data were collected |
| 4c | | How and where and participants were recruited |
| 4d | | Recruitment cost |
Definition:
Costs of recruitment (by source) and indication of how many participants were recruited by each source

## Slide 31
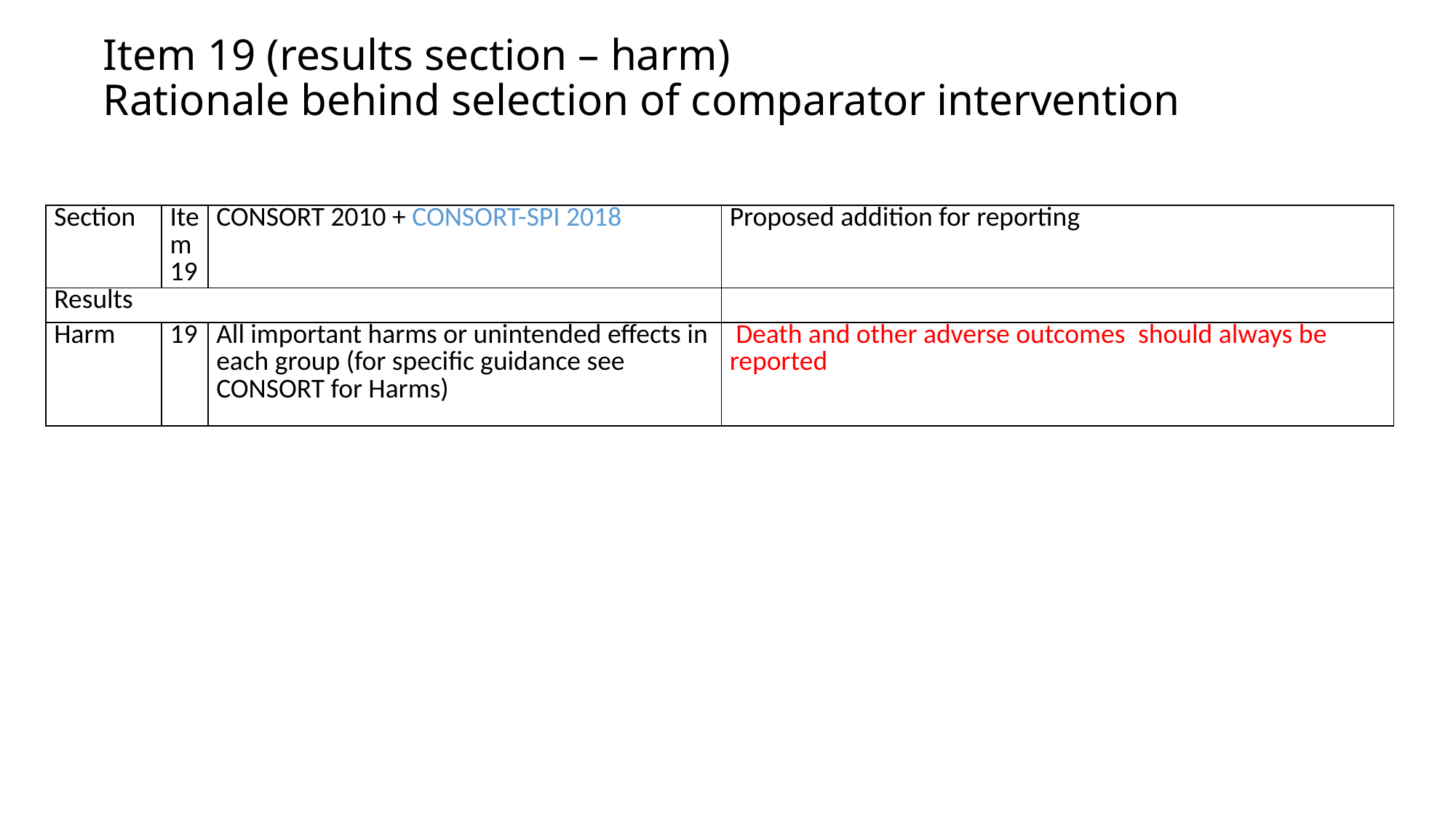

# Item 19 (results section – harm) Rationale behind selection of comparator intervention
| Section | Item 19 | CONSORT 2010 + CONSORT-SPI 2018 | Proposed addition for reporting |
| --- | --- | --- | --- |
| Results | | | |
| Harm | 19 | All important harms or unintended effects in each group (for specific guidance see CONSORT for Harms) | Death and other adverse outcomes  should always be reported |

## Slide 32
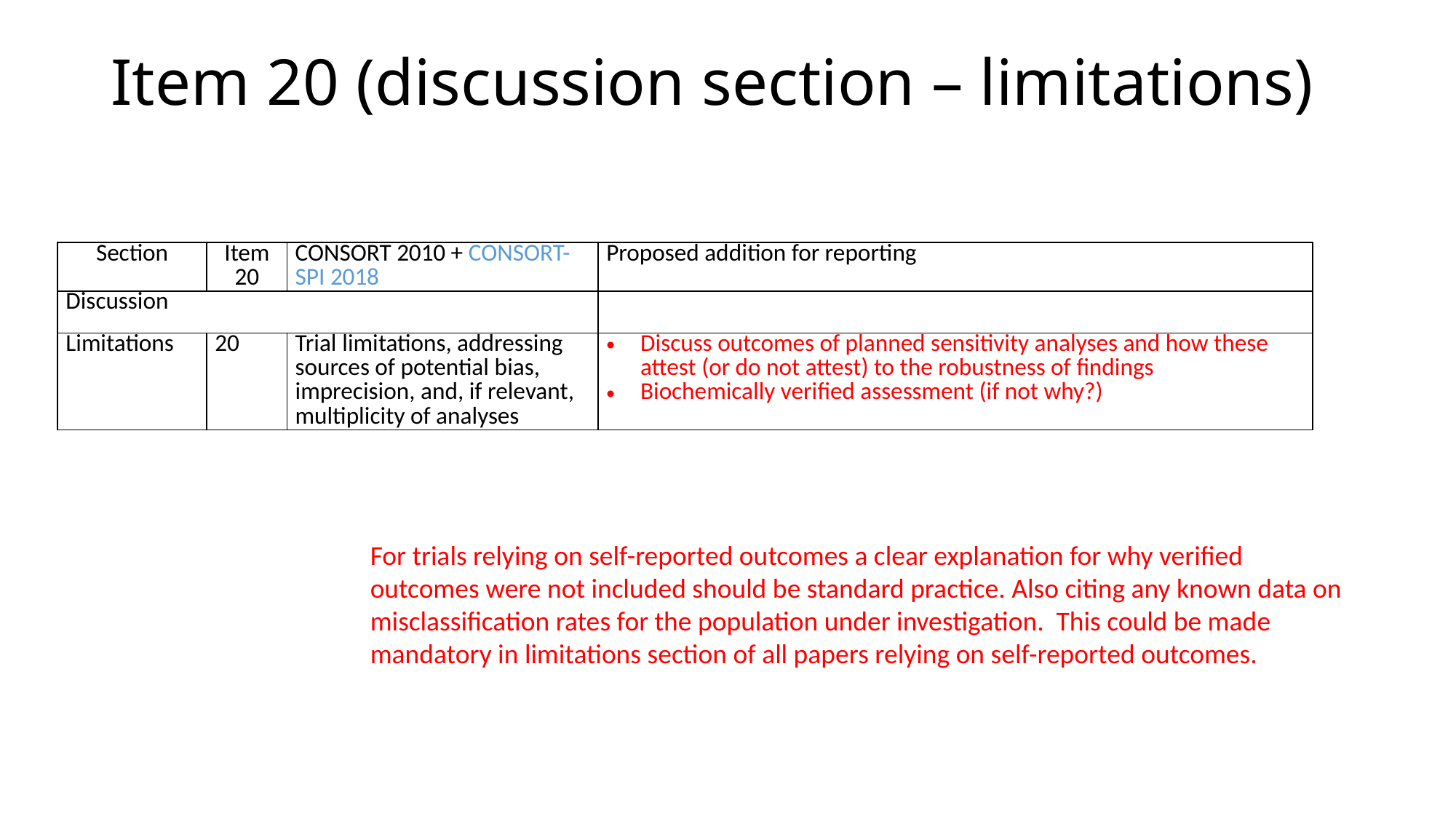

# Item 20 (discussion section – limitations)
| Section | Item 20 | CONSORT 2010 + CONSORT-SPI 2018 | Proposed addition for reporting |
| --- | --- | --- | --- |
| Discussion | | | |
| Limitations | 20 | Trial limitations, addressing sources of potential bias, imprecision, and, if relevant, multiplicity of analyses | Discuss outcomes of planned sensitivity analyses and how these attest (or do not attest) to the robustness of findings Biochemically verified assessment (if not why?) |
For trials relying on self-reported outcomes a clear explanation for why verified outcomes were not included should be standard practice. Also citing any known data on misclassification rates for the population under investigation. This could be made mandatory in limitations section of all papers relying on self-reported outcomes.

## Slide 33
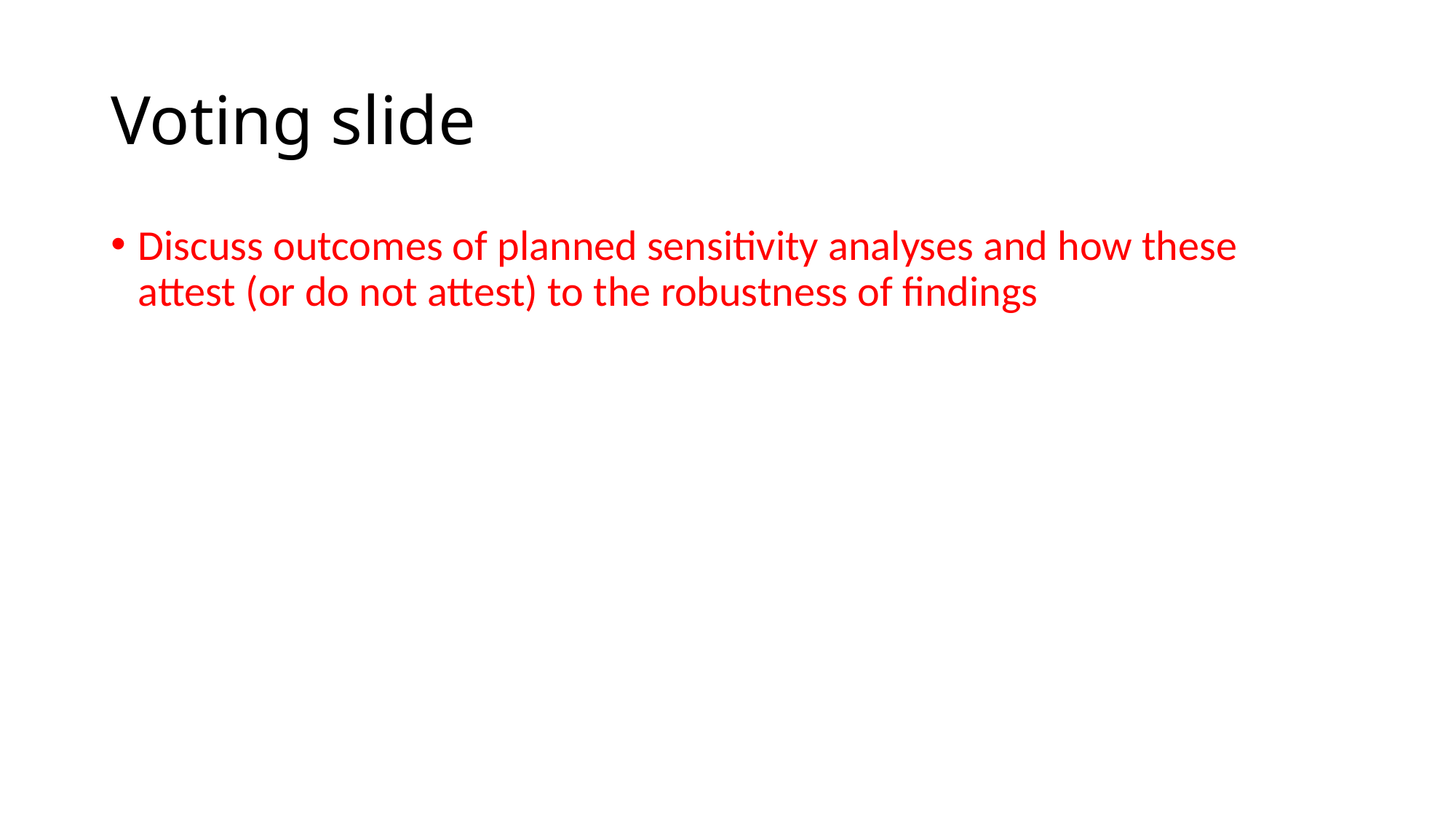

# Voting slide
Discuss outcomes of planned sensitivity analyses and how these attest (or do not attest) to the robustness of findings

## Slide 34
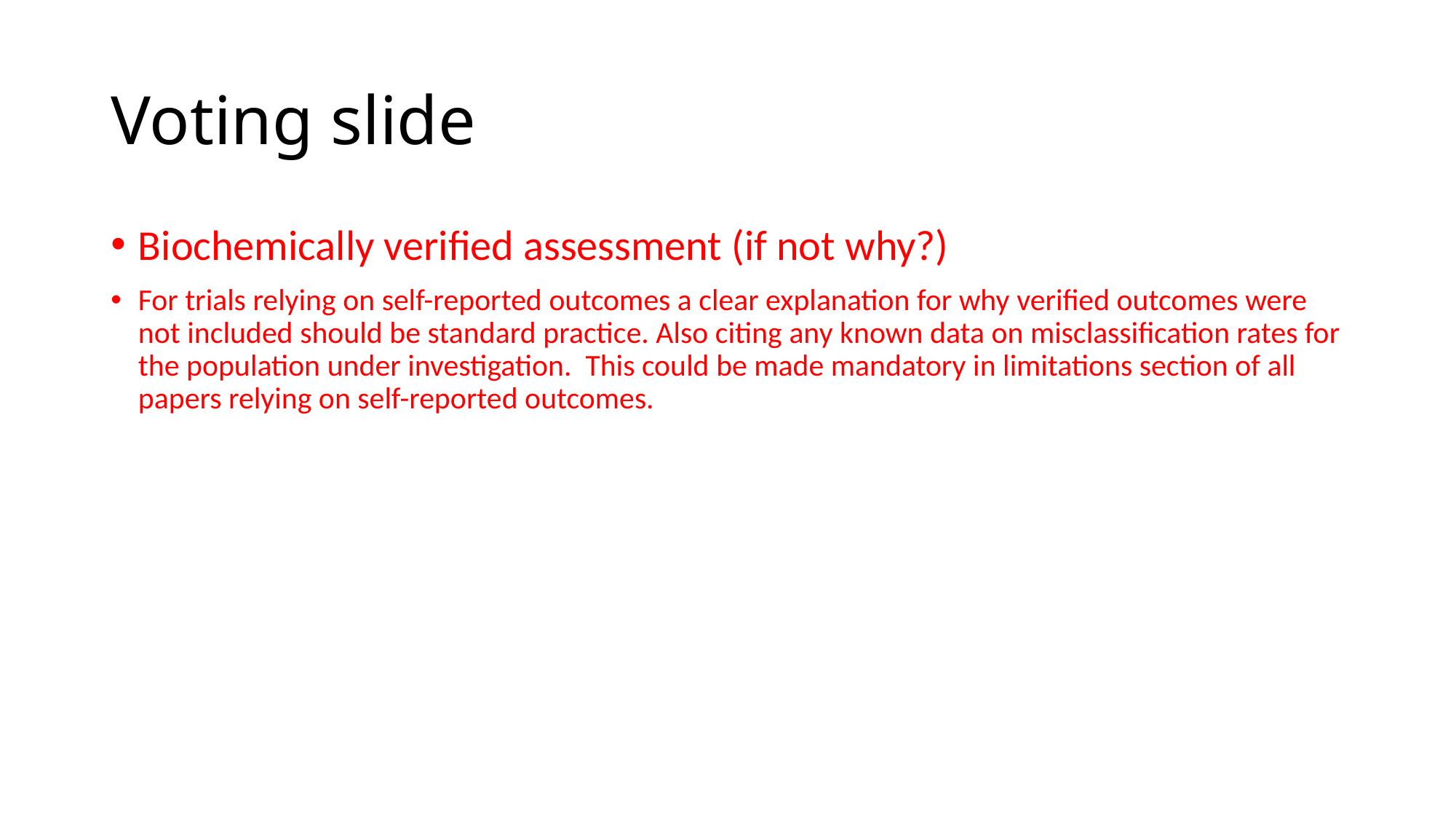

# Voting slide
Biochemically verified assessment (if not why?)
For trials relying on self-reported outcomes a clear explanation for why verified outcomes were not included should be standard practice. Also citing any known data on misclassification rates for the population under investigation. This could be made mandatory in limitations section of all papers relying on self-reported outcomes.
